# Functional analysis of microRNA and transcription factor synergistic regulatory network based on identifying regulatory motifs in non-small cell lung cancer

**DOI:** 10.1186/1752-0509-7-122

**Published:** 2013-11-07

**Authors:** Kening Li, Zihui Li, Ning Zhao, Yaoqun Xu, Yongjing Liu, Yuanshuai Zhou, Desi Shang, Fujun Qiu, Rui Zhang, Zhiqiang Chang, Yan Xu

**Affiliations:** 1 College of Bioinformatics Science and Technology, Harbin Medical University, Harbin 150081, China; 2 Institute of System Engineering, Harbin University of Commerce, Harbin 150028, China

**Keywords:** Regulatory network, MicroRNA, Transcription factor, Motif, Cell cycle, miR-17 family, Non-small cell lung cancer

## Abstract

**Background:**

Lung cancer, especially non-small cell lung cancer, is a leading cause of malignant tumor death worldwide. Understanding the mechanisms employed by the main regulators, such as microRNAs (miRNAs) and transcription factors (TFs), still remains elusive. The patterns of their cooperation and biological functions in the synergistic regulatory network have rarely been studied.

**Results:**

Here, we describe the first miRNA-TF synergistic regulation network in human lung cancer. We identified important regulators (MYC, NFKB1, miR-590, and miR-570) and significant miRNA-TF synergistic regulatory motifs by random simulations. The two most significant motifs were the co-regulation of miRNAs and TFs, and TF-mediated cascade regulation. We also developed an algorithm to uncover the biological functions of the human lung cancer miRNA-TF synergistic regulatory network (regulation of apoptosis, cellular protein metabolic process, and cell cycle), and the specific functions of each miRNA-TF synergistic subnetwork. We found that the miR-17 family exerted important effects in the regulation of non-small cell lung cancer, such as in proliferation and cell cycle regulation by targeting the retinoblastoma protein (RB1) and forming a feed forward loop with the E2F1 TF. We proposed a model for the miR-17 family, E2F1, and RB1 to demonstrate their potential roles in the occurrence and development of non-small cell lung cancer.

**Conclusions:**

This work will provide a framework for constructing miRNA-TF synergistic regulatory networks, function analysis in diseases, and identification of the main regulators and regulatory motifs, which will be useful for understanding the putative regulatory motifs involving miRNAs and TFs, and for predicting new targets for cancer studies.

## Background

Lung cancer, predominantly non-small cell lung cancer (NSCLC), is a common cause of malignant tumor death worldwide [1]. Since the end of the 20th century, lung cancer has become the leading cause of malignant tumor death, with morbidity and mortality gradually increasing over the past 50 years. Active and passive tobacco smoking is the best-known risk factor for lung cancer development.

Recent advances in genomics, epigenomics, transcriptomics, and molecular pathology, as well as in the sequencing techniques, have led to the identification of many potential factors as biomarkers, which may provide possibilities for the early detection of lung cancer and personalized therapy [2]. Several genes were identified as predictive biomarkers in NSCLC, such as the somatic mutation and gene copy gain of the epidermal growth factor receptor (EGFR) [3]. L-myc is amplified and expressed in human small cell lung cancer [4]. Although the oncogenicity of lung cancer-related genes has been studied extensively, there is limited knowledge of the process of malignant transformation and the regulatory mechanisms of multistep pathogenesis, especially the regulatory network of lung cancer-related genes, which urgently need to be studied [5].

MicroRNAs (miRNAs) are small non-coding RNAs (~23 nt long) that regulate gene expression at the post-transcriptional level. MiRNAs are encoded by genomic DNA, transcribed by RNA polymerase II and then incorporated into a RNA-induced silencing complex that binds to the 3′-UTR regions of its target mRNAs to repress translation or enhance degradation [6]. In recent years, important roles for miRNAs were identified in developmental timing, tumorigenesis, cell proliferation, and cell death [6,7]. MiRNAs function as oncogenes and tumor suppressors, and their regulatory effects in lung cancer development and progression have been demonstrated [8-10].

Hsa-let-7a acts as a protective miRNA that suppresses RAS and other transcriptional factors. Hsa-let-7a expression is generally reduced in NSCLC patients [11,12]. High expression of hsa-miR-155 was reported to be associated with poor survival in lung cancer patients [13]. Hsa-miR-128b directly regulates epidermal growth factor receptor (EGFR), and loss of heterozygosity of hsa-miR-128b was detected frequently in NSCLC patients [14]. Higher tumor miR-92a-2* levels are associated with decreased survival in patients with small cell lung cancer. MiRNAs can act as biomarkers of human lung cancer, and this may have important clinical applications in prognosis prediction and in predicting the molecular pathogenesis of cancer, as well as in the development of targeted therapies [15-17].

At the transcriptional level, transcription factors (TFs) are the main regulators that control the transcription of their target genes by binding to specific DNA sequences in the promoter regions of the genes. TFs and miRNAs are the two largest families of trans-acting, gene regulatory molecules in multicellular organisms, and they share a common regulatory logic [18]. Most genes in a genome are regulated not by a single factor, but instead by a synergistic network of trans-acting factors. At the network level, motifs comprising miRNAs, TFs, and common target genes were found to be widespread in diverse organisms from bacteria to human, suggesting that these motifs serve as basic building blocks of transcription networks. In our work, we have used the term “motif” to describe a small group that illustrates the regulation patterns of a miRNA, a TF, and their target genes. Common motifs, such as feedforward loops (FFLs) and feedback loops (FBLs), have been found to play crucial roles in gene regulation, such as the miR-17 cluster, the E2F1, and the c-Myc that modulates cellular proliferation in cancer [19]. Several databases of TF-miRNA FFLs involved in tumors have been developed [20,21]. Moreover, protein-protein interactions data have been included to construct regulatory networks for identifying novel regulatory motifs, such as the four or more node FFLs [22,23].

Previous studies into the co-regulation between miRNAs and TFs found a variety of significant network motifs that were over-represented in the co-regulation network [24,25], suggesting that the gene regulation system requires close synergistic regulation by transcriptional and post-transcriptional layers. However, the miRNA-TF synergistic effect may not be limited only to the FFLs. Non-loop forms, such as the cascaded form, which have also helped in understanding the regulatory mechanism, should be considered. Therefore, in this study, we have identified multiple types of motifs, including FFLs, miRNA- or TF-mediated FBLs, and miRNA or TF-mediated cascaded patterns.

Here, we used comprehensive data sources and algorithms to predict regulatory relationships of miRNAs and their targets in an attempt to provide the first miRNA-TF-mediated regulatory network in lung cancer. We also identified synergistic motifs of miRNAs and TFs. Several potential major factors were identified in subnetworks. We have developed an algorithm to predict the biological functions involved in the human lung cancer miRNA-TF regulatory network as well as the specific functions regulated by each synergistic motif. Our results showed that miRNAs of the same family exhibited similar regulatory modes, implying that miRNA family members tended to work together, particularly in regulating TFs. The miR-17 family (in an FFL with the E2F1and the RB1) was found to be an important family in the lung cancer regulation network.

This study provides a framework for constructing a lung cancer-related synergistic regulatory network and for analyzing the biological functions of the network. This approach could be applied easily to study other cancers, and may provide useful information for laboratory experiments and target validation.

## Results

### MiRNA and TF synergistic regulatory network in lung cancer

We collected and curated 1990 human lung cancer-related genes from several disease-related gene databases and 1823 genes that were aberrantly expressed in NSCLC samples. From them, we selected a total of 1002 genes that met the requirements of lung cancer-related genes to use in this study. The 100 bootstrapping repetitions that we conducted on the microarray data showed that the overlap between gene sets calculated based on the re-sampled data and the original gene set (1002 genes) was quite significant, suggesting that our selected lung cancer-related genes were robust. The ratios of overlap genes to original genes are listed in Additional file 1: Table S1.

By combining multiple algorithms, we obtained the targets of all the human miRNAs and TFs, and then used hypergeometric tests to obtain 252 lung cancer-related miRNAs and 173 TFs. Based on the relationships between lung cancer miRNAs/TFs and lung cancer genes, we constructed a lung cancer miRNA-TF synergistic regulatory network. The numbers of nodes and regulatory relations in the network are listed in Table 1.

**Table 1:** Summary of relationships in the lung cancer-related synergistic regulatory network

**Relationship**	**No. of pairs**	**No. of miRNAs**	**No. of genes**	**No. of TFs**
miRNA-gene^a^	29877	252	928	-
miRNA-TF^b^	1107	243	-	27
TF-gene^c^	1588	-	457	174
TF-miRNA^d^	207	93	-	56

^a^miRNA repression of gene expression.

^b^miRNA repression of TF expression.

^c^TF regulation of gene expression.

^d^TF regulation of miRNA expression.

**Figure 1:**
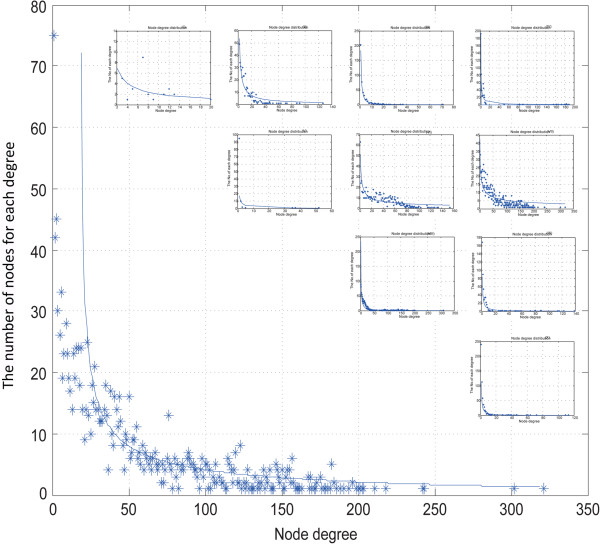
**Node degree distribution of the whole network and 10 subnetworks.** The X axis is the degree of a node, and the Y axis shows the number of nodes that correspond to the degree. The 10 small figures are for subnetworks I to X in order. The big figure is for the whole synergistic regulatory network.

The results of the node degree distribution analysis showed that most nodes had low degrees and only a few nodes had high degrees (Figure 1), which reflected a scale-free network. Therefore, hub nodes might play major roles in the synergistic regulatory network. Because the edges in the networks had directions, we identified the hub nodes with the highest incoming and outgoing degrees. As shown in Tables 2 and 3, eight of the top 10 TFs with higher outgoing degrees [26-35] and more than half of the hub nodes were either well-known lung cancer regulators, such as MYC and TP53, or related to lung cancer development and progression, such as E2F1 and SP1 [13,36-49]. This finding was a preliminary reflection of the robustness of the network. Notably, four of the top 10 hub miRNAs belonged to the miR-17 family, namely has-miR-106a/106b/20a/20b, indicating that these miR-17 family members are vital regulators in the regulatory network of human lung cancer. The top 5% of the hub TFs and miRNAs are shown in Additional file 1: Table S2. Some of the hubs listed in Tables 2 and 3 did not meet the enrichment test of hypergeometric cumulative distribution in 1000 randomization tests, suggesting that the hubs were caused by biological significance rather than by false-positive miRNA target data.

**Table 2:** Top 10 miRNAs and TFs with highest out-degree in lung cancer synergistic regulatory network

**Top**	**Regulator**	**Out- degree**	**Supported by**^**a**^
1	hsa-miR-590-3p	320	-
2	hsa-miR-548c-3p	302	-
3	hsa-miR-570	243	-
4	hsa-miR-340	242	-
5	hsa-miR-495	218	-
6	hsa-miR-106a^b^	207	PMID: 19209007
7	hsa-miR-106b^b^	202	-
8	hsa-miR-20a^b^	201	PMID: 16266980
9	hsa-miR-20b^b^	200	-
10	hsa-miR-944	200	-
1	MYC	116	PMID: 11720740
2	TP53	95	PMID: 12619108
3	E2F1	73	PMID: 22803943
4	TFAP2A	64	PMID: 22143938
5	SP1	61	PMID: 22158040
6	JUN	59	-
7	E2F4	50	PMID: 19473719
8	HIF1A	48	PMID: 22115707
9	NFKB1	48	-
10	STAT1	44	PMID: 17348819

^a^Supported by: published articles in which the TF or miRNA was experimentally verified as being related to lung cancer development and progression.

^b^belongs to the miR-17 family.

**Table 3:** Top 10 genes, TFs, and miRNAs with highest in-degree in lung cancer synergistic regulatory network

**Top**	**Gene**	**In- degree**	**Supported by**^**a**^
1	NTRK2	153	PMID: 21466358
2	ACVR2B	146	-
3	PLAG1	142	-
4	RAPH1	131	-
5	IGF1R	127	PMID: 22133293
6	CLCN5	123	-
7	FOXP1	123	PMID: 22904134
8	ACSL4	120	-
9	WHSC1	120	PMID: 22028615
10	ABHD2	120	-
1	E2F3	112	PMID: 16938365
2	ESR1	110	PMID: 18949413
3	PPARA	79	-
4	SMAD7	75	PMID: 21221812
5	ETS1	68	PMID: 17785952
6	MAFG	62	-
7	ETS2	56	PMID: 21922129
8	ARNT	54	PMID: 22645320
9	AHR	53	PMID: 21646808
10	FUBP1	51	PMID: 19258502
1	hsa-miR-129-5p	7	-
2	hsa-miR-19b	7	-
3	hsa-miR-219-5p	7	PMID: 16530703
4	hsa-miR-92a	7	-
5	hsa-miR-301b	6	-
6	hsa-miR-433	6	-
7	hsa-miR-557	6	-
8	hsa-miR-152	5	-
9	hsa-miR-16	5	PMID: 19549910
10	hsa-miR-329	5	-
11	hsa-miR-429	5	PMID: 19759262

^a^Supported by: published articles in which the gene, TF, or miRNA was experimentally verified as being related to lung cancer development and progression.

### Synergistic motif identification and subnetwork construction

**Figure 2:**
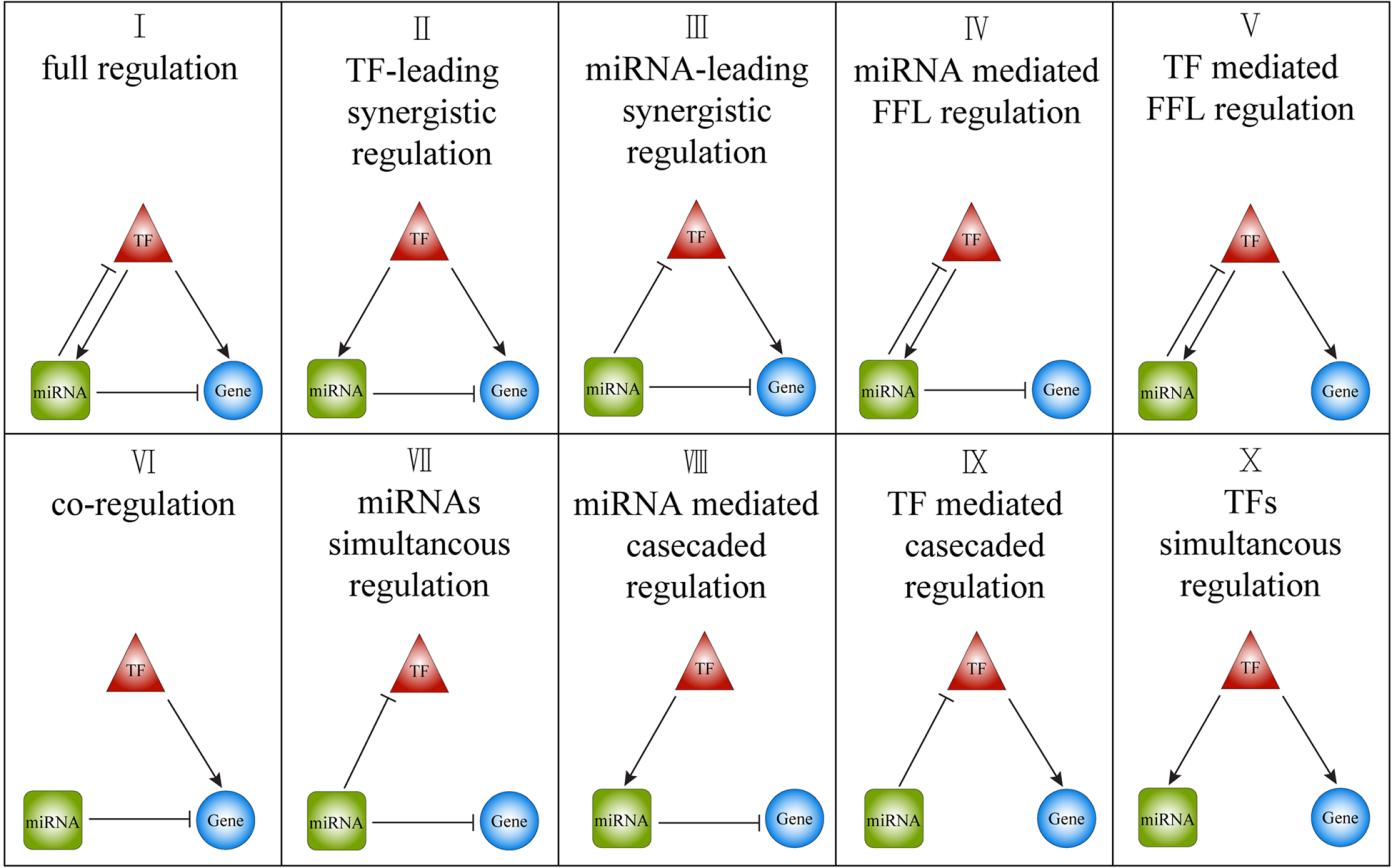
**The 10 kinds of motifs identified in this study.** The ellipse nodes are the genes; the round rectangle nodes are the miRNAs; and the triangle nodes are the TFs.

In the human lung cancer synergistic regulatory network, we identified a total of eight types of synergistic motifs consisting of a miRNA, a TF, and their synergistically regulated target genes, including full regulation, miRNA- or TF-leading synergistic regulation, miRNA- or TF-mediated FFL regulation, co-regulation, and miRNA- or TF-mediated cascade regulation. We also identified two other kinds of regulatory motifs, namely, miRNA simultaneous regulation and TF simultaneous regulation (Figure 2). To evaluate the significance of the synergistic motifs, we ran 10000 random simulations (see Methods). The results of P-values indicated that eight of the observed motifs differed significantly from the results expected by chance (see Table 4 for details). To rank the motif types, we also calculated Z scores for them. The synergistic regulatory motifs with the highest Z scores were co-regulation and TF-mediated cascade regulation types; all were in non-loop formation and all comprised regulatory relations that were derived from miRNAs. The motifs with the lowest Z scores were Motifs X and V, and they were the only two non-significant motifs with P-values greater than 0.01.

**Table 4:** Details of motifs in the lung cancer synergistic regulatory network

**Motif**	**Z-value**^**a**^**/P-value**^**b**^	**Mean**^**c**^	**Std**^**c**^	**No. of motifs**	**No. of genes**	**No. of TFs**	**No. of miRNA**
I	4.9847/ 0	27.0475	11.8268	86	28	3	12
II	6.9558/ 0	1417.3280	81.6113	1985	180	22	219
III	2.4034/ 0.0097	438.9193	30.8235	513	176	35	66
IV	4.2270/ 0.0001	771.7246	260.5343	1873	520	4	13
V	2.3031/ 0.0162	230.3356	87.9953	433	97	4	13
VI	19.9562/ 0	32689.19	290.5763	38488	422	163	250
VII	27.6201/ 0	114787.3	1225.9080	148647	928	26	243
VIII	2.9824/ 0.0004	19895.26	369.7436	20995	882	56	88
IX	8.3071/ 0	12070.81	267.1441	14290	237	27	242
X	1.2440/ 0.108	4284.108	155.8588	4478	414	56	88

^a^Z-value was calculated using the formula (2.4.1).

^b^P-value is the proportion of the 10000 random simulations in which a motif had a larger frequency in the random repeats than real in the data.

^c^Mean and Std are the average and the standard deviation of motif frequency of the 10000 random repeats.

Motif I: Full regulation; II: TF-leading synergistic regulation; III: miRNA leading synergistic regulation; IV: miRNA feedback synergistic regulation; V: TF feedback synergistic regulation; VI: synergistic co-regulation; VII: miRNA simultaneous regulation; VIII: linear regulation from TF; IX: linear regulation from miRNA; and X: TF simultaneous regulation.

**Figure 3:**
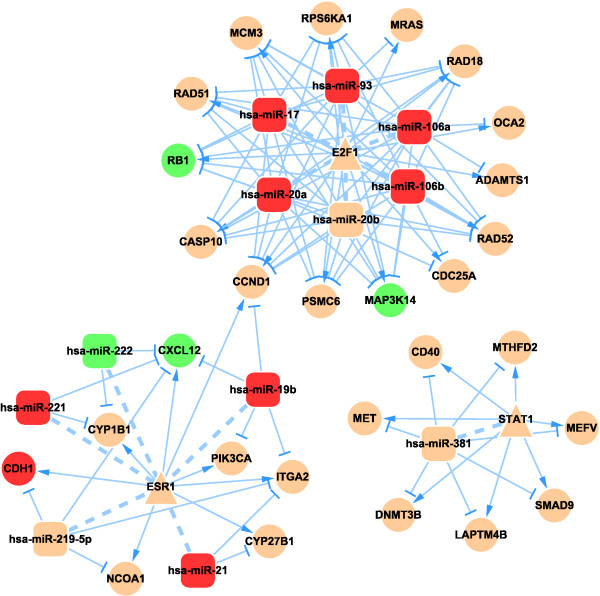
**Lung cancer-related miRNA-TF synergistic regulatory subnetwork I.** The ellipse nodes are the genes; the round rectangle nodes are the miRNAs; and the triangle nodes are the TFs. Green nodes: down-regulated nodes; red nodes: up-regulated nodes; arrow shape edge: transcriptional activation/repression; T-shape edge: miRNA repression; and dash line: a feedback loop.

We performed manual literature mining with the combined keywords “miRNA&TF&cancer” to confirm the relationship between the motifs and lung cancer or other types of cancers. Names of the motif components (gene, TF, or miRNA) and “prognosis&cancer” were combined as keywords to search for motifs that had predictive power for prognosis (Table 5). Because all the motifs were identified in this search, we merged motifs of the same type into a subnetwork, and consequently obtained 10 subnetworks, which are presented in Figure 3 (subnetwork I) and which are available in Cytoscape format in the Additional file 2. Based on the motif type, we named the subnetworks I to X to reflect the motif names. To find the hub regulators under each regulatory motif type, we analyzed the degree distributions of the 10 subnetworks. All the subnetworks had the features of a scale-free network as shown in Figure 1. Therefore, we extracted the hub nodes of each factor type in all the subnetworks according to the criteria discussed in the Methods section. The results are listed them in Additional file 1: Table S3. To determine the distribution of the regulators among the 10 subnetworks, we counted the number of motifs that each regulator participated in (Additional file 1: Table S4). We found that each TF was involved in an average of 2.377 motifs and 43.7% of them were in motifs above the average, while each miRNA was involved in an average of 4.89 motifs and 34.1% of them were in motifs above the average. Notably, three TFs (STAT1, E2F1, and ESR10) participated in all motifs and seven miRNAs participated in all motifs, namely hsa-miR-106a, hsa-miR-20a, hsa-miR-17, hsa-miR-19b, hsa-miR-381, hsa-miR-21, and hsa-miR-221. The first four of these miRNAs belong to the miR-17 family, further indicating the important role of the miR-17 family in the network.

**Table 5:** Examples of motifs or prognosis components of motifs

**Motif**	**Example**	**Supported by**^**a**^	**Prognosis**	**Supported by**	**P-value**^**b**^
I	miR-106a& E2F1	PMID: 18521848	miR-106a &E2F1 &RAD51	PMID:20219352&16166473 &15956972	0.03790692
II	miR-27b& ESR1	-	miR-181a &TP53 &RUNX3	PMID:20363096&17401424 &15819721	0.001892851
III	miR-16& MYC	PMID: 22002311	miR-16 &JUN &LPL	PMID:21400525&9484827 &21508119	3.311478e-06
IV	miR-106a& E2F1	PMID: 22002038	miR-17 &STAT1 &ALDH1A3	PMID:22065543&20581241 &22960273	1.80563e-21
V	miR-17& E2F1	PMID: 18171346	miR-21 &ESR1 &CXCL12	PMID:20508945&20109227	0.006603943
VI	miR-548& MYC	-	Let-7d &ATF1 &GSTP1	PMID:21725603&22631637 &22045684	3.35018e-07
VII	miR-20b& ESR1	PMID: 22002038	miR-200c &E2F3 &ALDH1A3	PMID:20579395&15122326 &23436614	6.592982e-22
VIII	miR-152& POU2F1	PMID: 21712563	miR-141 &SOX2 & CXCL12	PMID:21445232&20532662 &16631235	1.471662e-21
IX	miR-19a& ESR1	PMID: 20080637	CTNNB1 & miR-21 & SMAD7	PMID:17949785&20508945 &12584741	0.0002268908
X	miR-34c-5p& MYC	PMID: 22585994	GADD45A& miR-34 & P53	PMID:12171872&19736307 &22978804	2.885798e-06

^a^Supported by: published articles in which the gene, TF, or miRNA was experimentally verified as working together or have a prognosis function.

^b^P-value: the P-value of hypergeometric cumulative distribution to test whether the motifs were enriched with gene mutations.

### Biological functions of the synergistic regulatory networks and subnetworks

First, mutations in the genes of ten subnetworks were analyzed. We downloaded somatic mutation profiles of 538 lung adenocarcinomas (LUAD) and 178 lung squamous cell carcinomas (LUSC) from The Cancer Genome Atlas level 2 data. Then, we selected genes in each subtype with mutation rates greater than 5% as a mutated gene set. Hypergeometric cumulative distribution was used to test the enrichment significance for genes in 10 kinds of motifs. The results showed that the 10 kinds of motifs were all significantly enriched in the mutated gene set.

After obtaining all the synergistic motifs in the human lung cancer regulatory network, we next examined the Gene Ontology (GO) biological process (BP) terms and the Kyoto Encyclopedia of Genes and Genomes (KEGG) pathways they regulated. We developed an algorithm to analyze the network functions based on the results of the BP and pathway enrichment analyses (see Methods). All enriched terms were scored and sorted in descending order, and the top 5% were defined as common terms. A total of 36 common GO terms were identified and clustered under the following functions: regulation of apoptosis and programmed cell death, regulation of cellular protein and phosphate metabolic process, receptor protein signaling pathway, regulation of protein phosphorylation and modification process, cell cycle, regulation kinase activity, DNA repair, and metabolic process (Table 6). The 22 common KEGG pathways that were identified were similar to the common BP terms (Table 7). The common pathways were ranked according to their scores from high to low. The top 10 pathways included P53 pathway, direct P53 effectors, regulation of telomerase, and cell cycle; and the lowest ranked pathways included E2F transcription factor network and canonical Wnt signaling pathway ranked 20 and 22, respectively (Table 7).

**Table 6:** Biology process terms regulated by the miRNA-TF synergistic regulatory network

**GO term**	**Annotation**	**Rank**^**a**^	**In motifs**^**b**^
GO:0042981	Regulation of apoptosis	1	10
GO:0032268	Regulation of cellular protein metabolic process	2	10
GO:0007167	Enzyme linked receptor protein signaling pathway	3	9/I
GO:0031399	Regulation of protein modification process	4	10
GO:0042325	Regulation of phosphorylation	5	10
GO:0019220	Regulation of phosphate metabolic process	6	10
GO:0051329	Interface of mitotic cell cycle	7	10
GO:0000082	G1/S transition of mitotic cell cycle	8	9/IV
GO:0001932	Regulation of protein phosphorylation	9	10
GO:0007169	Transmembrane receptor protein tyrosine kinase signaling pathway	10	9/I
GO:0071156	Regulation of cell cycle arrest	11	9/IV
GO:0045859	Regulation of protein kinase activity	12	10
GO:0000075	Cell cycle checkpoint	13	9/IV
GO:0006259	DNA metabolic process	14	10
GO:0006281	DNA repair	15	9/IV
GO:0043549	Regulation of kinase activity	16	10
GO:2000045	Regulation of G1/S transition of mitotic cell cycle	17	9/IV
GO:0000084	S phase of mitotic cell cycle	18	9/IV
GO:0051320	S phase	19	9/IV
GO:0007093	Mitotic cell cycle checkpoint	20	9/IV
GO:0031575	Mitotic cell cycle G1/S transition checkpoint	21	8/III, IV
GO:0071779	G1/S transition checkpoint	22	8/III, IV
GO:0006468	Protein phosphorylation	23	9/I
GO:0043066	Negative regulation of apoptosis	24	9/I
GO:0043069	Negative regulation of programmed cell death	25	9/I
GO:0031328	Positive regulation of cellular biosynthetic process	26	9/I
GO:0071900	Regulation of protein serine/threonine kinase activity	27	9/I
GO:0009968	Negative regulation of signal transduction	28	9/I
GO:0048011	Nerve growth factor receptor signaling pathway	29	9/I
GO:0046777	Protein autophosphorylation	30	7/I, II, V
GO:0006355	Regulation of transcription, DNA-dependent	31	9/I
GO:2001141	Regulation of RNA biosynthetic process	32	9/I
GO:0009967	Positive regulation of signal transduction	33	8/I, V
GO:0006357	Regulation of transcription from RNA polymerase II promoter	34	9/I
GO:0043065	Positive regulation of apoptosis	35	9/I
GO:0045893	Positive regulation of transcription, DNA-dependent	36	8/I,III

^a^Rank: is the rank number calculated using the formula (2.7.1) based on the number of occurrences of the GO terms among all the assigned terms.

^b^In motifs: is how many motif types (subnetworks) were assigned the corresponding GO term. The roman number(s) following the slash indicate the subnetwork(s) in which the corresponding GO term was not found.

**Table 7:** Pathways regulated by miRNA-TF synergistic regulatory network

**Pathway name**	**Rank**^**a**^	**In motifs**^**b**^
p53 pathway	1	8
Direct p53 effectors	2	8
Regulation of Telomerase	3	7
Hypoxia and oxygen homeostasis regulation of HIF-1-alpha	4	8
Arf6 signaling events	5	8
Cell Cycle, Mitotic	6	7
S Phase	7	6
Synthesis of DNA	8	7
DNA Replication	9	6
Regulation of DNA replication	13	5
G1/S Transition	10	6
IGF1 pathway	11	6
Orc1 removal from chromatin	12	5
Switching of origins to a post-reflective state	14	5
Removal of licensing factors from origins	15	5
Signaling events regulated by Ret tyrosine kinase	16	4
EphrinA-EPHA pathway	17	3
E-cadherin signaling events	18	7
FOXA transcription factor networks	19	6
E2F transcription factor network	20	7
Neurotrophic factor-mediated Trk receptor signaling	21	9
Canonical Wnt signaling pathway	22	7

^a^Rank: is the rank number calculated using the formula (2.7.1) based on the number of occurrences of the pathways among all the assigned pathways.

^b^In motifs: is how many motif subnetworks were assigned the corresponding pathways.

We speculated whether the specific BP terms for each subnetwork were regulated specifically by each motif type. After removing the 36 common BP terms, the remaining terms were ranked by their enrichment frequency in the subnetworks and then categorized within each subnetwork. Details of the results are shown in Additional file 1: Table S5. The functions of the miRNAs in the lung cancer regulatory network were predicted from subnetwork VII because it comprised motifs with miRNAs that simultaneously regulated TFs and genes, while TFs that regulated genes or miRNAs were not included in these motifs. The predicted functions were regulation of fibroblast growth factor signaling pathway, inositol lipid-mediated signaling, response to insulin stimulus, MAPK cascade, receptor signaling pathway, cell migration, DNA replication and metabolism, phosphorylation, enzymatic activity, and meiosis. Similarly, the functions of the TFs were predicted from subnetwork X; they included regulation of protein metabolic process, apoptosis and programmed cell death, gene expression, phosphorylation, and regulation of enzyme activity. Each motif in subnetwork I comprised a FBL and FFL, and not surprisingly, their specific function was DNA replication, which requires precise and complex regulation because of its ubiquity in cells and the multiple enzymes involvement.

### Interplay of miRNA and TF in the human lung cancer regulatory network

Of the 252 miRNAs in the regulatory network, 93 (36.9%) were regulated by TFs. Most of these miRNAs had low in-degree, and only 11.8% (11 of the 93) had in-degrees greater than 5. Of the 173 TFs in the network, 27 (15.6%) were regulated by miRNAs, and 37.0% (10 of 27) of them had in-degrees greater than 50. A total of 57 TFs regulated miRNAs and 244 miRNAs regulated TFs. On average, each TF was regulated by 9.33 miRNAs, while each miRNA was regulated by 1.86 TFs. By comparing the intensity and density of the interplay between the miRNAs and TFs in the lung cancer regulatory network, we found that only a small number of the TFs were regulated by miRNAs at a high intensity, while most miRNAs were regulated by TFs at a significantly lower intensity (Additional file 1: Figure S11).

**Figure 4:**
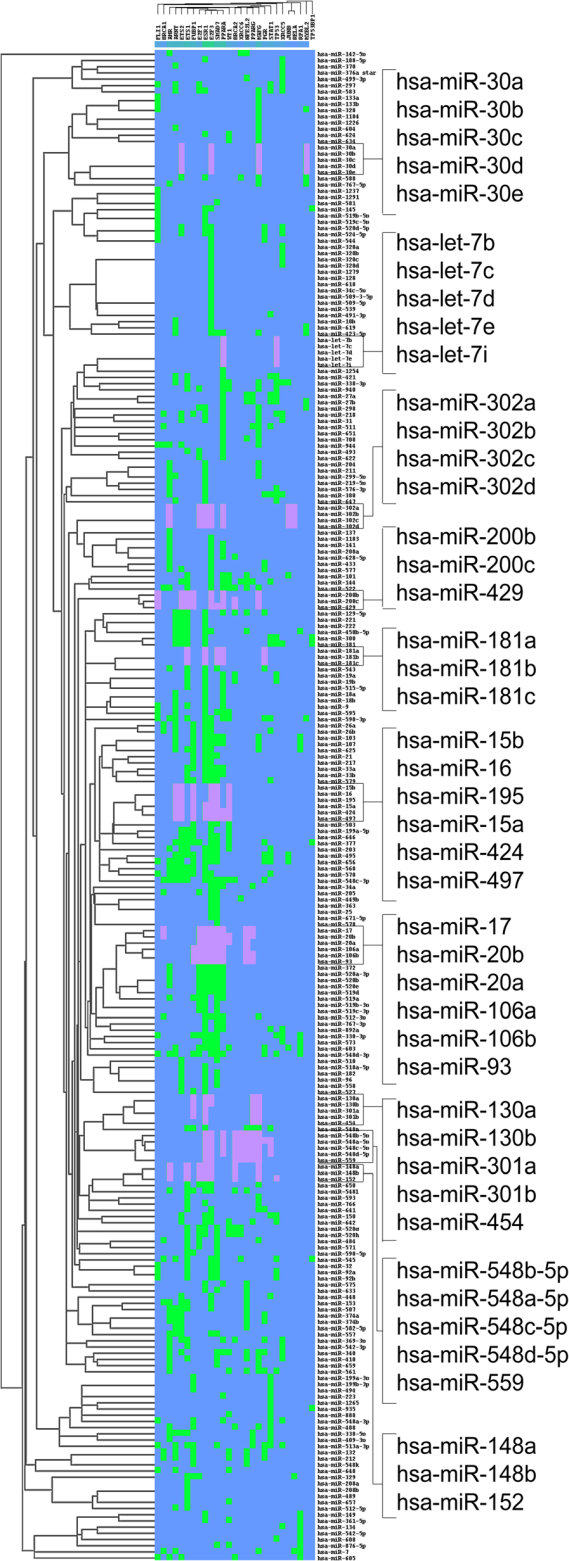
**Heatmap of miRNA-TF hierarchical clustering.** Green: miRNAs regulated by TFs; blue: non-regulatory relations; dark pink: regulations to TFs of miRNAs that were clustered closely in the hierarchical tree and belonged to a same family. Square brackets: zoom in view of the miRNAs on the left of the figure. MiRNAs in square brackets belonged to different families.

In some subnetworks, the miRNAs that belonged to the same family tended either to function together or to synergistically regulate targets. To further clarify this observation, we performed a two-way clustering analysis based on the regulatory relations between the 252 miRNAs and 173 TFs. We found that 10 miRNA families involving 45 miRNAs were clustered and that the miRNAs from one family had similar target TFs (Figure 4).

### A model of the miR-17 family, RB1, and E2F1 motif in lung cancer proliferation

In subnetwork I, we discovered the predicted interactions between the miR-17 family and E2F1 for the first time (Additional file 1: Table S6). Interestingly, six members of the miR-17 family (miR-17/20a/20b/106a/106b/93) clustered in one group. The miR-17 family and E2F1 formed a FBL, which was a clique. A similar phenomenon was reported for the miR-17-92 cluster (miR-17/20a/18a/19a/19b-1/92-1), which forms a FBL with E2F1, and plays roles in regulating cellular proliferation and apoptosis. The interactions between the miR-17-92 cluster and E2F1 have been verified experimentally [50-54]. The miR-17 family and the miR-17-92 cluster have two shared members, miR-17 and miR-20a, both of which were confirmed to interact with E2F1. For the other four members of the miR-17 family, we performed a sequence alignment to examine how likely they were to interact with E2F1. The conserved sequence of E2F1 among five species (*Homo sapiens*, *Mus musculus*, *Pan troglodytes, Rattus norvegicus and Bos taurus*) was aligned to the mature sequences of the six members of the miR-17 family. All miR-17 family members shared 8–15 bases with the E2F1 conserved sequence (Additional file 1), and their interaction was predicted by at least five algorithms (Additional file 1: Table S6), supporting the high possibility of an interaction between the miR-17 family and E2F1.

The RB1 tumor suppressor negatively regulates the cell cycle and is inactivated in a wide range of human tumors [55]. In subnetwork I, RB1 was targeted by members of the miR-17 family (Additional file 1: Table S6) and E2F1, while the miR-17 family members and E2F1 targeted each other, thereby forming an FFL. By analyzing the miRNA expression profiles of NSCLC patients, we found that five miR-17 family members were significantly overexpressed, the expression of RB1 was significantly down-regulated, and E2F1 expression was not significantly different (Additional file 1: Table S7). MiR-20b was the exception to this because no probe was detected. Next, we examined the mechanism by which the miR-17 family regulates cell cycle and tumor progression in lung cancer using a hypothetical model. The interaction between the pRb proteins and the E2F TF family plays a central role in regulating cell cycle progression by controlling the expression of E2F-dependent cell cycle genes [56]. The overexpressed miR-17 family may directly decrease the translation of RB1, thereby lowering the expression of the RB1 protein. In G0 or early G1 cells, Rb protein, which has been functionally inactivated by transcriptional suppression, releases the transactivation domains of E2F and activates the expression of genes that encode products necessary for S-phase progression [50]. Moreover, E2F1 promotes the transcription of the miR-17 family, which causes overexpression of the miR-17 family members, thereby governing cell cycle and proliferation of lung tumors by targeting RB1 protein.

## Discussion

Here we constructed the first lung cancer-related miRNA-TF synergistic regulatory network of lung cancer. We identified 10 types of motifs and constructed 10 subnetworks. More than half of the putative hub nodes were verified by examining other published works, which indicated the robustness of the network. We developed an algorithm to understand the common and specific functions of these networks. Finally, we proposed a hypothetical model to explain the role of the miR-17 family in regulating cell cycle and tumor progression by targeting the RB1 protein in NSCLC.

In the human miRNA-TF synergistic regulatory network and subnetworks, hub genes and hub miRNAs were identified. Most either were known lung cancer-related factors or were reported to play important roles in lung cancer. The hubs with highest out-degrees in the regulatory network, Myc, TP53, and E2F1, are TFs that play roles in apoptosis, cell proliferation, and lung tumor development. The amplification and overexpression of Myc has been detected in lung cancer of different histologic subtypes [26]. TP53 encodes tumor protein p53, abnormalities of which are frequently found in lung cancers [27]. E2F1 overexpression was reported produce more aggressive tumors with a high proliferation rate during the progression of NSCLC [57]. MiR-17/106a/20a/93/34a were the hubs of many subnetworks, and four of them belong to the miR-17 family. MiR-17 and miR-20a were reported to induce apoptosis in lung cancer cells [35] and miR-34 s was found to be dramatically down-regulated in NSCLC [58].

In this work, we proposed a model to predict the regulatory role of the miR-17 family in the cell cycle via RB1 and E2F1. In the model, five out of six miR-17 family members were significantly overexpressed in NSCLC cells where they enhanced the repression of the RB1 gene, which is responsible for the G1 checkpoint and blockage of S-phase entry and cell growth. Hesan et al. [59] confirmed the up-regulation of four members of the miR-17 family in colorectal carcinoma tissues and showed that they promote cell proliferation and tumor growth by targeting the RND3 tumor suppressor gene. A similar group, the miR-17-92 cluster with two members that were common with the miR-17 family, had diverse functions in the regulation of cellular differentiation, proliferation, and apoptosis. The two common members, miR-17 and miR-20a, were shown to temper an E2F1-induced G1 checkpoint to regulate cell cycle progression [50]. Furthermore, the E2F and the miR-17-92 cluster could form FBLs [51], and in the cancer regulation network, FBLs involving miR-17-92, E2F and MYC have been reported [52]. We checked the interactive relations of the miR-17 family with E2F1 and RB1 by sequence alignment and found a strong possibility of their interactions. Moreover, many regulatory relationships support our predictive model of the miR-17 family, E2F1, and RB1 motif, which demonstrates the effectiveness of our regulatory network.

After identifying the miRNA-TF synergistic motifs, we calculated their significance and Z-values, and ranked the motifs according to their Z scores. The first-ranked Motif VII was more significant than the second-ranked Motif VI, possibly because of the availability of abundant miRNA regulation data but insufficient TF regulation data. One reason that Motif VI was found to be the most significant regulatory motif in the network may be that genes are first regulated by TFs at the transcription level and then by miRNAs at the post-transcription level; thus, genes are significantly regulated by TFs and miRNAs separately at different times and in different locations in the cell. By comparing two Motifs, II and III, and by examining the regulatory directions between the miRNAs and TFs, we found that miRNAs tended to be significantly regulated by TFs rather than regulate TFs. This observation is despite the fact that data on the - targets of TFs are limited, while much more data on the targets of miRNAs are available. Therefore, we inferred that TFs play a dominant role in FFL regulation. This assumption is supported by the results of another study, which found that TFs held dominant positions in the global regulatory system (i.e. at the transcriptional level) compared with the miRNAs at the downstream positions (i.e. at the post-transcriptional level) [23]. Between the linear motifs (VIII, IX), Motif IX was more significant than Motif VIII, which indicated that genes tended to be regulated directly by TFs, while miRNAs tended to regulate TFs while they were being formed rather than act as a mediated regulator between TFs and their target genes.

The expression level analysis of genes and miRNAs may help in understanding the regulatory mechanisms; therefore, the differentially expressed genes in our networks further investigated. In general, we found that up-regulated miRNAs down-regulated their target genes by degrading them at the transcript level or by repressing protein production at the translational level. We also observed the reverse, in which down-regulated miRNAs led to up-regulated target genes. However, down-regulated miRNAs that down-regulated their target genes and up-regulated miRNAs that up-regulated their target genes were also observed in our network. This may be because the expression levels of genes or miRNAs are determined by multiple factors, including environment, heredity, copy number variations, and epigenetics. Thus, miRNAs and TFs may influence expression to a great degree rather than being the decisive factors.

In this study, we analyzed the regulation of genes by miRNAs and TFs, but did not consider gene-to-gene relationships. Cui et al. [60,61] studied the relationship among oncogenes in the context of activity/inhibitory motifs and compared the number of mutant genes and miRNA target genes in each type of motif. In the future, we will examine the activation, inhibition, and physical interactions among the genes in regulatory networks, and discuss the regularity role of miRNAs, TFs, and motifs. Our future studies will contribute to uncovering the principles of miRNA regulation in signal transduction networks.

## Conclusions

In summary, our established miRNA and TF synergistic regulatory network in NSCLC has provided clues about the regulatory mechanisms of lung cancer and information that will help identify the core regulators. Nearly half of the hub regulators, as well as the proposed regulatory motifs, were confirmed by literature searches, which indicated the effectiveness and rationality of the network construction. The most significant motifs were of the co-regulation and TF-mediated cascade regulation types. While cooperating with miRNAs, TFs tended to play a dominant role in FFL regulations. We also developed an algorithm to analyze the functions of the human lung cancer miRNA-TF regulatory network and subnetworks. According to the full regulation subnetwork and expression analysis, we proposed a predictive model of the miR-17 family, E2F1 and RB1 in the regulation of cell cycle and cellular proliferation. Our study will provide valuable information for lung cancer investigators to identify critical elements and regulatory motifs for a better understanding of the regulatory mechanisms or for designing future experimental studies of lung cancer.

## Methods

### Lung cancer-related genes

**Figure 5:**
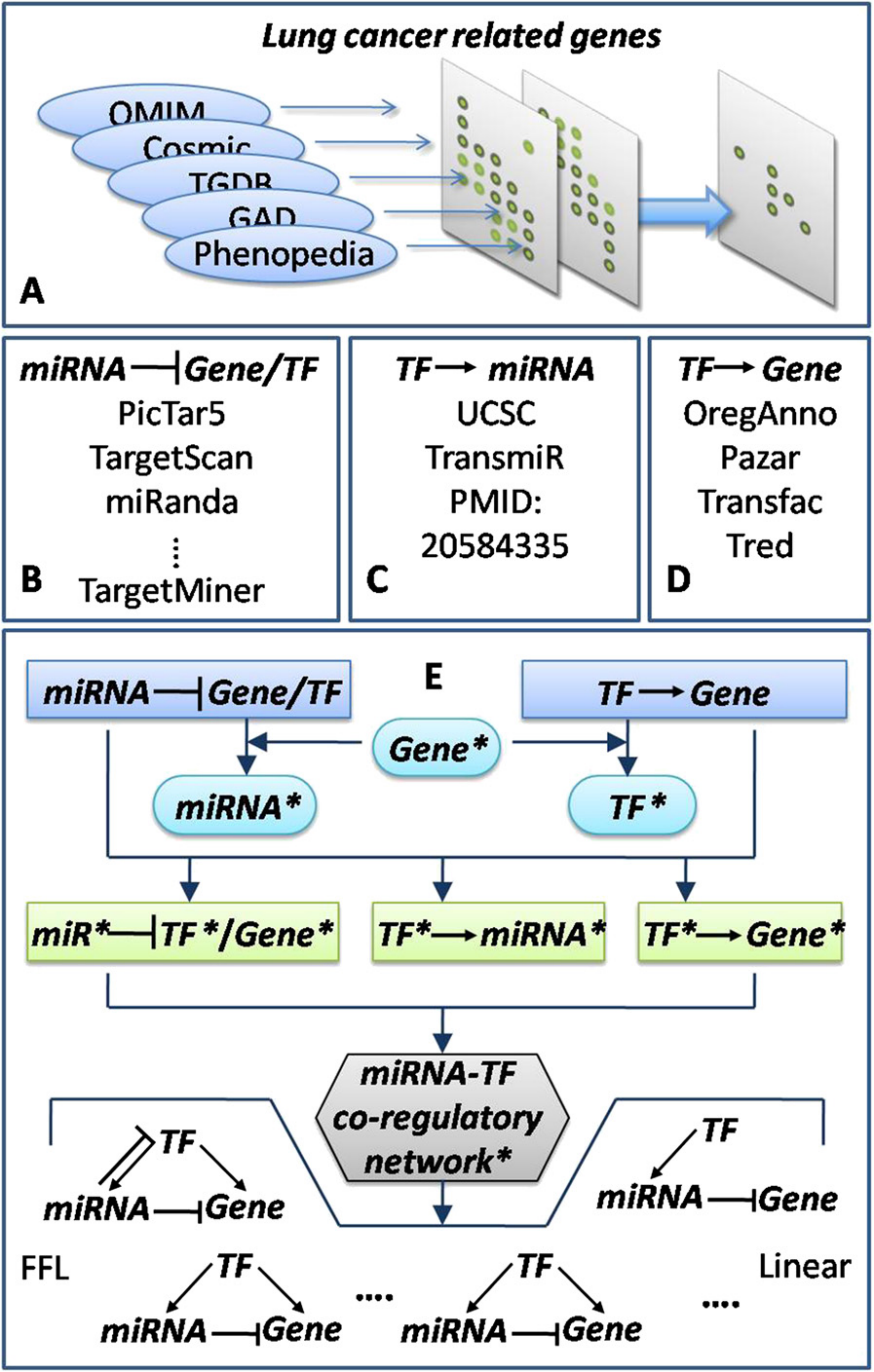
**Workflow of data collection, miRNA-TF synergistic regulatory network construction, and motif identification. A**: Lung cancer-related gene collection. Lung cancer genes were obtained from five databases, differential expressed genes in two microarray datasets were calculated, and the overlapped set were used in the present study. **B**, **C**, and **D** showed the data source or algorithms of regulator-target relations. **E**: Workflow of synergistic regulatory network construction and motif identification. Elements with a star mark '*’ are 'lung cancer related-’. First, by Gene^*^ from step A and miRNA target relations from step B, we obtained miRNA^*^ using a hypergeometric test. After a similar procedure for getting TF^*^, we combined any two of Gene^*^, miRNA^*^, and TF^*^with their regulatory relations to obtain four types of regulatory relation^*^. Then, we merged them to construct the miRNA-TF synergistic regulatory network^*^. Last, 10 motif types were identified.

Lung cancer-related genes were collected using an integrated strategy as follows: 1) We retrieved published genes with somatic mutation or vital for lung cancer from five well-established cancer- and disease-related gene databases, namely Phenopedia [62], Cosmic [63], GAD [64], TGDB, and OMIM [65]. 2) Aberrantly expressed genes were obtained from two gene expression profiles of NSCLC samples published in the NCBI GEO database [66], namely, [GEO:GSE2088] and [GEO:GSE11969]. In the profile GSE2088, 48 squamous cell carcinoma samples, nine adenocarcinoma samples and 30 normal samples were investigated. We used only 125 NSCLC samples from the profile GSE11969, including 35 squamous cell carcinoma samples, 90 adenocarcinoma samples, and five normal samples, and ignored samples with other subtype. We carried out a profile preprocessing step on the samples, which included filtering out data with more than 5% missing values, combining probes of the same gene, and then filling missing values using the K-neighbors algorithm. We screened differentially expressed genes using the criteria of fold change value > 1.5 and false discovery rate (FDR) < 0.01. Differentially expressed genes that were common to the two profiles were included. 3) We selected the intersection of the two gene sets obtained from the two previous steps to use in the present study (Figure 5A). Because gene lists can change when re-sampling is applied to microarray data [67], we conducted a bootstrapping procedure with 100 replications on the microarray data to confirm the robustness of the selected lung cancer-related genes.

### Target prediction of miRNAs and TFs

For miRNA target prediction, we combined 10 popular databases or algorithms, namely, miRanda, TargetScan, PicTar5, PITA, DIANA-microT, mirSVR, RNA22, RNAhybrid, MirTarget2, and TargetMiner (Additional file 1: Table S8). To decrease the number of false-positive results, only miRNA–mRNA interactions predicted by at least three of the algorithms were accepted as positive. TFs were treated as genes when predicting miRNA–TF regulations.

TFs and their experimentally proven targets were retrieved from four databases: ORegAnno [68], Pazar [69], Transfac [70], and Tred [71]. To ensure that the results were complete, we used a union set of all the retrieved data.

MiRNA precursor sequences were obtained from the miRBase database. We selected the 2-kb upstream regions of the pre-miRNAs as their putative promoter regions, and then searched the sequences for TF binding sites using the UCSC genome browser (Z score =2.33) [72]. The selected sites were required to be conserved among human, rat, and mouse. We also incorporated experimentally supported TF–miRNA regulatory relations that were curated manually from large numbers of published papers [25] and from TransmiR [21] (Figure 5C).

### Significant lung cancer-related miRNAs and TFs

After obtaining the miRNA/TF and targets relations, we calculated the regulators whose targets were significantly enriched with lung cancer genes using the hypergeometric cumulative distribution function followed by the Benjamini & Hochberg adjustment procedure. The cutoff value was set to 0.01.

(1) $$P=1-\sum_{i=0}^{x}\frac{\left(\begin{array}{l} k\\ i \end{array}\right)\;\left(\begin{array}{l} M‒k\\ N‒i \end{array}\right)}{\left(\begin{array}{l} M\\ N \end{array}\right)}$$

where, M is the total number of all human genes in the human genome, k is the number of lung cancer-related genes, N is the number of target genes for a certain miRNA (or TF), and x is the number of target genes of a certain miRNA (or TF) that overlapped with lung cancer-related genes. To avoid the effect from false-positives in the miRNA target data, we conducted randomization tests to ensure the biological significance of the identified lung cancer-related miRNAs and TFs. Instead of using 1002 lung cancer-related genes to enrich each of the miRNAs and TFs, we randomly selected 1002 genes from all the coding genes in the human genome for enrichment, and this procedure was repeated 1000 times.

### Motif identification and statistics test

Based on the regulatory relationships that we predicted, network motifs of miRNA-TF synergistically regulatory patterns were identified, including the FFLs and non-loop form (Figure 5E). We performed statistical analysis to estimate the significance of each motif type. Specifically, in random networks, each node maintained the number of incoming and outgoing edges that they had in the corresponding node in the real network. Then, the number of each type of motifs was counted, and this was repeated 10000 times. The significance value (P-value) of one motif type indicates the proportion of the 10000 repeats when the motif was observed in the random networks was no less than its appearance in the real lung cancer synergistic regulatory network.

(2) $$P=\frac{N_{\mathrm{high}}}{N_{\mathrm{random}}}$$

where, *N*_*high*_ is the number of random times that an acquired motif was more than or equal to the real network, and *N*_*random*_ is 10000.

We also calculated Z scores for all the motif types to estimate by how many standard deviations an observation was above or below the mean.

(3) $$Z=\frac{N_{\mathrm{real}}-N_{\mathrm{mean}}}{\mathrm{SD}}$$

where, *N*_*real*_ and *N*_*mean*_ are the number of motifs observed in the real synergistic regulatory network and their mean occurrence in the random networks, respectively. SD is the standard deviation of the number of motifs in the random networks.

### Motif-specific subnetwork and hub definition

All network motifs of the same type were merged to construct a motif-specific subnetwork (for example, all FFLs were merged to form the FFL subnetwork) and then the subnetworks were visualized by the Cytoscape software [73]. When network sizes were greatly different from one another, we defined hub nodes as the top 5% highest-degree nodes of the miRNAs, TFs, and genes in both the subnetwork and whole network.

### Expression level analysis of miRNAs, TFs, and genes

MiRNA and gene expression profiles were downloaded from the NCBI Gene Expression Omnibus (GEO), and differentially expressed genes were calculated using the significance analysis of microarrays (SAM) software [74] with the FDR set to less than 1%. The union set of differentially expressed genes in the GSE2088 and GSE11969 datasets was used to determine the lung cancer-related genes, while the intersection was shown in different colors in the motif-specific subnetwork. The miRNA expression profiles from the GSE27705 dataset, which included 20 NSCLC samples and 10 normal samples, were used to identify up- or down-regulated expressed miRNAs.

### BP and pathway analysis of the genes in the human lung cancer synergistic regulatory network and synergistic subnetworks

For the function analysis of each subnetwork, we developed an algorithm to obtain the most representative functions among the numerous GO BP terms and KEGG pathways after BP and pathway enrichment analysis. For example, for the BP function analysis of subnetwork_i_ (Motif_i_) the algorithm: 1) found the target gene set shared by each miRNA-TF pair, which contained no less than three genes; 2) all target gene sets were enriched with BP terms by the hypergeometric test with P-value adjustment by FDR (the cutoff of P-value was 0.005); and 3) the frequency that each BP term was enriched was counted and represented as *Frequent*_*i*_ (i∈[1,10]), and all enriched terms were ranked in descending order according to their frequency value, so that the rank number of each BP term, represented as *Motif*_*i*_*-rank*, could be obtained.

Terms with high commonality existed in most subnetworks and ranked high in each subnetwork and, therefore, could be assumed to represent the main functions of the network. The commonality score of each BP term was calculated as

(4) $$\sum_{i=1}^{10}\frac{\mathrm{Frequen}t_{i}}{\mathrm{moti}f_{i}-\mathrm{rank}*{\mathrm{motif}}_{i}-\mathrm{stagenumber}}$$

where, stagenumber is the number of stages in motif_i_. The terms with the highest top 5% score were regarded as common terms, and were used to represent the functions of the whole regulatory network. We then removed the common terms from each subnetwork and ranked the remaining terms to identify the specific functions of each subnetwork. Pathway function analysis was conducted using the same procedure.

### Hierarchical clustering and sequence alignment

In our analysis of the interplay between miRNAs and TFs, two-way clustering of the regulators was performed using Cluster 3.0 software [75] and the resultant heatmap was viewed using TreeView. MiRNA family information was obtained from miRBase [76]. Multiple sequences were aligned using ClustalW2 software in the analysis tools framework at EMBL-EBI [77].

## Competing interests

The authors declare that they have no competing interests.

## Authors’ contributions

XY designed the study, LK, LZ and ZN performed the research, LY, ZY, QF and ZR analyzed the data, and LZ and LK wrote the manuscript. All authors have read and approved the final version.

## Authors’ information

Kening Li, Zihui Li and Ning Zhao *considered as co-first authors.*

## Supplementary Material

Additional file 1: Table S1 Is provided as the ratio of overlap genes and original genes after bootstrappings; **Table S2** is the hub TFs and miRNAs of lung cancer synergistic regulatory network; **Table S3** is the hub miRNAs and TFs of subnetwork Ito X; **Table S4** is the count of motif types (subnetworks) miRNAs or TFs belong to; **Table S5** shows specific functions of miRNA-TF regulatory subnetwork Ito X; **Table S6** indicates target genes (E2F1 and RB1) predictive results of the miR-17 family; **Table S7** is provided as differential expression analysis of the miR-17 family and RB1 by SAM; **Table S8** is a list of miRNA-target relation predictive algorithms and databases used in our work. Click here for file

Additional file 2 miRNA-TF synergetic regulatory subnetwork I to X in order. Click here for file

## References

[B1] Siegel R Naishadham D Jemal A Cancer statistics, 2012 CA: a cancer journal for clinicians 2012 62 10 29 10.3322/caac.20138 22237781

[B2] Chen HY Yu SL Li KC Yang PC Biomarkers and transcriptome profiling of lung cancer Respirology 2012 17 620 626 10.1111/j.1440-1843.2012.02154.x 22372638

[B3] Dahabreh IJ Linardou H Siannis F Kosmidis P Bafaloukos D Murray S Somatic EGFR mutation and gene copy gain as predictive biomarkers for response to tyrosine kinase inhibitors in non-small cell lung cancer Clin Cancer Res 2010 16 291 303 10.1158/1078-0432.CCR-09-1660 20028749

[B4] Nau MM Brooks BJ Battey J Sausville E Gazdar AF Kirsch IR McBride OW Bertness V Hollis GF Minna JD L-myc, a new myc-related gene amplified and expressed in human small cell lung cancer Nature 1985 318 69 73 10.1038/318069a0 2997622

[B5] Ye H Liu X Lv M Wu Y Kuang S Gong J Yuan P Zhong Z Li Q Jia H MicroRNA and transcription factor co-regulatory network analysis reveals miR-19 inhibits CYLD in T-cell acute lymphoblastic leukemia Nucleic Acids Res 2012 40 5201 5214 10.1093/nar/gks175 22362744 PMC3384304

[B6] Ambros V The functions of animal microRNAs Nature 2004 431 350 355 10.1038/nature02871 15372042

[B7] Schickel R Boyerinas B Park SM Peter ME MicroRNAs: key players in the immune system, differentiation, tumorigenesis and cell death Oncogene 2008 27 5959 5974 10.1038/onc.2008.274 18836476

[B8] Zhang B Pan X Cobb GP Anderson TA microRNAs as oncogenes and tumor suppressors Dev Biol 2007 302 1 12 10.1016/j.ydbio.2006.08.028 16989803

[B9] Lu J Getz G Miska EA Alvarez-Saavedra E Lamb J Peck D Sweet-Cordero A Ebert BL Mak RH Ferrando AA MicroRNA expression profiles classify human cancers Nature 2005 435 834 838 10.1038/nature03702 15944708

[B10] Khoshnaw SM Green AR Powe DG Ellis IO MicroRNA involvement in the pathogenesis and management of breast cancer J Clin Pathol 2009 62 422 428 10.1136/jcp.2008.060681 19398594

[B11] Grosshans H Johnson T Reinert KL Gerstein M Slack FJ The temporal patterning microRNA let-7 regulates several transcription factors at the larval to adult transition in C. elegans Dev Cell 2005 8 321 330 10.1016/j.devcel.2004.12.019 15737928

[B12] Takamizawa J Konishi H Yanagisawa K Tomida S Osada H Endoh H Harano T Yatabe Y Nagino M Nimura Y Reduced expression of the let-7 microRNAs in human lung cancers in association with shortened postoperative survival Cancer Res 2004 64 3753 3756 10.1158/0008-5472.CAN-04-0637 15172979

[B13] Yanaihara N Caplen N Bowman E Seike M Kumamoto K Yi M Stephens RM Okamoto A Yokota J Tanaka T Unique microRNA molecular profiles in lung cancer diagnosis and prognosis Cancer cell 2006 9 189 198 10.1016/j.ccr.2006.01.025 16530703

[B14] Weiss GJ Bemis LT Nakajima E Sugita M Birks DK Robinson WA Varella-Garcia M Bunn PA Jr Haney J Helfrich BA EGFR regulation by microRNA in lung cancer: correlation with clinical response and survival to gefitinib and EGFR expression in cell lines Ann Oncol 2008 19 1053 1059 10.1093/annonc/mdn006 18304967

[B15] Bartels CL Tsongalis GJ MicroRNAs: novel biomarkers for human cancer Clin Chem 2009 55 623 631 10.1373/clinchem.2008.112805 19246618

[B16] Raponi M Dossey L Jatkoe T Wu X Chen G Fan H Beer DG MicroRNA classifiers for predicting prognosis of squamous cell lung cancer Cancer Res 2009 69 5776 5783 10.1158/0008-5472.CAN-09-0587 19584273

[B17] Yu SL Chen HY Chang GC Chen CY Chen HW Singh S Cheng CL Yu CJ Lee YC Chen HS MicroRNA signature predicts survival and relapse in lung cancer Cancer cell 2008 13 48 57 10.1016/j.ccr.2007.12.008 18167339

[B18] Hobert O Gene regulation by transcription factors and microRNAs Science 2008 319 1785 1786 10.1126/science.1151651 18369135

[B19] O'Donnell KA Wentzel EA Zeller KI Dang CV Mendell JT c-Myc-regulated microRNAs modulate E2F1 expression Nature 2005 435 839 843 10.1038/nature03677 15944709

[B20] El Baroudi M Cora D Bosia C Osella M Caselle M A curated database of miRNA mediated feed-forward loops involving MYC as master regulator PloS one 2011 6 e14742 10.1371/journal.pone.0014742 21390222 PMC3048388

[B21] Wang J Lu M Qiu C Cui Q TransmiR: a transcription factor-microRNA regulation database Nucleic acids Res 2010 38 D119 122 10.1093/nar/gkp803 19786497 PMC2808874

[B22] Sun J Gong X Purow B Zhao Z Uncovering MicroRNA and Transcription Factor Mediated Regulatory Networks in Glioblastoma PLoS Comput Biol 2012 8 e1002488 10.1371/journal.pcbi.1002488 22829753 PMC3400583

[B23] Lin CC Chen YJ Chen CY Oyang YJ Juan HF Huang HC Crosstalk between transcription factors and microRNAs in human protein interaction network BMC Syst Biol 2012 6 18 10.1186/1752-0509-6-18 22413876 PMC3337275

[B24] Chen CY Chen ST Fuh CS Juan HF Huang HC Coregulation of transcription factors and microRNAs in human transcriptional regulatory network BMC bioinformatics 2011 12 Suppl 1 S41 10.1186/1471-2105-12-S1-S41 21342573 PMC3044298

[B25] Qiu C Wang J Yao P Wang E Cui Q microRNA evolution in a human transcription factor and microRNA regulatory network BMC Syst Biol 2010 4 90 10.1186/1752-0509-4-90 20584335 PMC2914650

[B26] Zajac-Kaye M Myc oncogene: a key component in cell cycle regulation and its implication for lung cancer Lung Cancer 2001 34 Suppl 2 S43 46 11720740 10.1016/s0169-5002(01)00343-9

[B27] Toyooka S Tsuda T Gazdar AF The TP53 gene, tobacco exposure, and lung cancer Human mutation 2003 21 229 239 10.1002/humu.10177 12619108

[B28] Duan HY Cao JX Qi JJ Wu GS Li SY An GS Jia HT Cai WW Ni JH E2F1 enhances 8-chloro-adenosine-induced G2/M arrest and apoptosis in A549 and H1299 lung cancer cells Biochemistry Biokhimiia 2012 77 261 269 10.1134/S0006297912030042 22803943

[B29] Rauch TA Wang Z Wu X Kernstine KH Riggs AD Pfeifer GP DNA methylation biomarkers for lung cancer Tumour Biol 2012 33 287 296 10.1007/s13277-011-0282-2 22143938

[B30] Hsu TI Wang MC Chen SY Yeh YM Su WC Chang WC Hung JJ Sp1 expression regulates lung tumor progression Oncogene 2012 31 3973 3988 10.1038/onc.2011.568 22158040 PMC3432230

[B31] Bankovic J Stojsic J Jovanovic D Andjelkovic T Milinkovic V Ruzdijic S Tanic N Identification of genes associated with non-small-cell lung cancer promotion and progression Lung Cancer 2010 67 151 159 10.1016/j.lungcan.2009.04.010 19473719

[B32] Munksgaard Persson M Johansson ME Monsef N Planck M Beckman S Seckl MJ Ronnstrand L Pahlman S Pettersson HM HIF-2alpha expression is suppressed in SCLC cells, which survive in moderate and severe hypoxia when HIF-1alpha is repressed Am J Pathol 2012 180 494 504 10.1016/j.ajpath.2011.10.014 22115707

[B33] Li J Yu B Song L Eschrich S Haura EB Effects of IFN-gamma and Stat1 on gene expression, growth, and survival in non-small cell lung cancer cells J Interferon Cytokine Res 2007 27 209 220 10.1089/jir.2006.0111 17348819

[B34] Navarro A Marrades RM Vinolas N Quera A Agusti C Huerta A Ramirez J Torres A Monzo M MicroRNAs expressed during lung cancer development are expressed in human pseudoglandular lung embryogenesis Oncology 2009 76 162 169 10.1159/000201569 19209007

[B35] Hayashita Y Osada H Tatematsu Y Yamada H Yanagisawa K Tomida S Yatabe Y Kawahara K Sekido Y Takahashi T A polycistronic microRNA cluster, miR-17-92, is overexpressed in human lung cancers and enhances cell proliferation Cancer research 2005 65 9628 9632 10.1158/0008-5472.CAN-05-2352 16266980

[B36] Terry J De Luca A Leung S Peacock G Wang Y Elliot WM Huntsman D Immunohistochemical expression of neurotrophic tyrosine kinase receptors 1 and 2 in lung carcinoma: potential discriminators between squamous and nonsquamous subtypes Arch Pathol Lab Med 2011 135 433 439 21466358 10.5858/2010-0038-OA.1

[B37] Nakagawa M Uramoto H Oka S Chikaishi Y Iwanami T Shimokawa H So T Hanagiri T Tanaka F Clinical significance of IGF1R expression in non-small-cell lung cancer Clin Lung Cancer 2012 13 136 142 10.1016/j.cllc.2011.10.006 22133293

[B38] Feng J Zhang X Zhu H Wang X Ni S Huang J High expression of FoxP1 is associated with improved survival in patients with non-small cell lung cancer Am J Clin Pathol 2012 138 230 235 10.1309/AJCPDHQFNYJZ01YG 22904134

[B39] Toyokawa G Cho HS Masuda K Yamane Y Yoshimatsu M Hayami S Takawa M Iwai Y Daigo Y Tsuchiya E Histone lysine methyltransferase Wolf-Hirschhorn syndrome candidate 1 is involved in human carcinogenesis through regulation of the Wnt pathway Neoplasia 2011 13 887 898 22028615 10.1593/neo.11048 PMC3201566

[B40] Bandi N Zbinden S Gugger M Arnold M Kocher V Hasan L Kappeler A Brunner T Vassella E miR-15a and miR-16 are implicated in cell cycle regulation in a Rb-dependent manner and are frequently deleted or down-regulated in non-small cell lung cancer Cancer Res 2009 69 5553 5559 10.1158/0008-5472.CAN-08-4277 19549910

[B41] Gibbons DL Lin W Creighton CJ Rizvi ZH Gregory PA Goodall GJ Thilaganathan N Du L Zhang Y Pertsemlidis A Kurie JM Contextual extracellular cues promote tumor cell EMT and metastasis by regulating miR-200 family expression Genes & development 2009 23 2140 2151 10.1101/gad.1820209 19759262 PMC2751985

[B42] Cooper CS Nicholson AG Foster C Dodson A Edwards S Fletcher A Roe T Clark J Joshi A Norman A Nuclear overexpression of the E2F3 transcription factor in human lung cancer Lung Cancer 2006 54 155 162 10.1016/j.lungcan.2006.07.005 16938365

[B43] Suga Y Miyajima K Oikawa T Maeda J Usuda J Kajiwara N Ohira T Uchida O Tsuboi M Hirano T Quantitative p16 and ESR1 methylation in the peripheral blood of patients with non-small cell lung cancer Oncology Rep 2008 20 1137 1142 18949413

[B44] Li X Yang XX Hu NY Sun JZ Li FX Li M A risk-associated single nucleotide polymorphism of SMAD7 is common to colorectal, gastric, and lung cancers in a Han Chinese population Mol Biol Rep 2011 38 5093 5097 10.1007/s11033-010-0656-3 21221812

[B45] Yamaguchi E Nakayama T Nanashima A Matsumoto K Yasutake T Sekine I Nagayasu T Ets-1 proto-oncogene as a potential predictor for poor prognosis of lung adenocarcinoma Tohoku J Exp Med 2007 213 41 50 10.1620/tjem.213.41 17785952

[B46] Bai J Hu S Transcriptome network analysis reveals potential candidate genes for squamous lung cancer Int J Mol Med 2012 29 95 101 21922129 10.3892/ijmm.2011.796

[B47] Yan Z Shah PK Amin SB Samur MK Huang N Wang X Misra V Ji H Gabuzda D Li C Integrative analysis of gene and miRNA expression profiles with transcription factor-miRNA feed-forward loops identifies regulators in human cancers Nucleic acids Res 2012 40 e135 10.1093/nar/gks395 22645320 PMC3458521

[B48] Chiba T Uchi H Yasukawa F Furue M Role of the arylhydrocarbon receptor in lung disease Int Arch Allergy Immunol 2011 155 Suppl 1 129 134 21646808 10.1159/000327499

[B49] Singer S Malz M Herpel E Warth A Bissinger M Keith M Muley T Meister M Hoffmann H Penzel R Coordinated expression of stathmin family members by far upstream sequence element-binding protein-1 increases motility in non-small cell lung cancer Cancer Res 2009 69 2234 2243 10.1158/0008-5472.CAN-08-3338 19258502

[B50] Pickering MT Stadler BM Kowalik TF miR-17 and miR-20a temper an E2F1-induced G1 checkpoint to regulate cell cycle progression Oncogene 2009 28 140 145 10.1038/onc.2008.372 18836483 PMC2768269

[B51] Sylvestre Y De Guire V Querido E Mukhopadhyay UK Bourdeau V Major F Ferbeyre G Chartrand P An E2F/miR-20a autoregulatory feedback loop J Biol Chem 2007 282 2135 2143 17135249 10.1074/jbc.M608939200

[B52] Aguda BD Kim Y Piper-Hunter MG Friedman A Marsh CB MicroRNA regulation of a cancer network: consequences of the feedback loops involving miR-17-92, E2F, and Myc Proc Natl Acad Sci USA 2008 105 19678 19683 10.1073/pnas.0811166106 19066217 PMC2598727

[B53] Conkrite K Sundby M Mukai S Thomson JM Mu D Hammond SM MacPherson D miR-17 92 cooperates with RB pathway mutations to promote retinoblastoma Genes & development 2011 25 1734 1745 10.1101/gad.17027411 21816922 PMC3165937

[B54] Olive V Jiang I He L mir-17-92, a cluster of miRNAs in the midst of the cancer network Int J Biochem Cell Biol 2010 42 1348 1354 10.1016/j.biocel.2010.03.004 20227518 PMC3681296

[B55] Giacinti C Giordano A RB and cell cycle progression Oncogene 2006 25 5220 5227 10.1038/sj.onc.1209615 16936740

[B56] Macaluso M Montanari M Giordano A Rb family proteins as modulators of gene expression and new aspects regarding the interaction with chromatin remodeling enzymes Oncogene 2006 25 5263 5267 10.1038/sj.onc.1209680 16936746

[B57] Huang CL Liu D Nakano J Yokomise H Ueno M Kadota K Wada H E2F1 overexpression correlates with thymidylate synthase and survivin gene expressions and tumor proliferation in non small-cell lung cancer Clin Cancer Res 2007 13 6938 6946 10.1158/1078-0432.CCR-07-1539 18056168

[B58] Bommer GT Gerin I Feng Y Kaczorowski AJ Kuick R Love RE Zhai Y Giordano TJ Qin ZS Moore BB p53-mediated activation of miRNA34 candidate tumor-suppressor genes Curr Biol 2007 17 1298 1307 10.1016/j.cub.2007.06.068 17656095

[B59] Luo H Zou J Dong Z Zeng Q Wu D Liu L Up-regulated miR-17 promotes cell proliferation, tumour growth and cell cycle progression by targeting the RND3 tumour suppressor gene in colorectal carcinoma Biochem J 2012 442 311 321 10.1042/BJ20111517 22132820

[B60] Cui Q Ma Y Jaramillo M Bari H Awan A Yang S Zhang S Liu L Lu M O'Connor-McCourt M A map of human cancer signaling Mol Syst Biol 2007 3 152 18091723 10.1038/msb4100200 PMC2174632

[B61] Cui Q Yu Z Purisima EO Wang E Principles of microRNA regulation of a human cellular signaling network Mol Syst Biol 2006 2 46 16969338 10.1038/msb4100089 PMC1681519

[B62] Yu W Clyne M Khoury MJ Gwinn M Phenopedia and Genopedia: disease-centered and gene-centered views of the evolving knowledge of human genetic associations Bioinformatics 2010 26 145 146 64 10.1093/bioinformatics/btp618 19864262 PMC2796820

[B63] Forbes SA Bindal N Bamford S Cole C Kok CY Beare D Jia M Shepherd R Leung K Menzies A COSMIC: mining complete cancer genomes in the Catalogue of Somatic Mutations in Cancer Nucleic acids Res 2011 39 D945 950 10.1093/nar/gkq929 20952405 PMC3013785

[B64] Becker KG Barnes KC Bright TJ Wang SA The genetic association database Nat Genet 2004 36 431 432 10.1038/ng0504-431 15118671

[B65] Hamosh A Scott AF Amberger JS Bocchini CA McKusick VA Online Mendelian Inheritance in Man (OMIM), a knowledgebase of human genes and genetic disorders Nucleic acids Res 2005 33 D514 517 15608251 10.1093/nar/gki033 PMC539987

[B66] Barrett T Wilhite SE Ledoux P Evangelista C Kim IF Tomashevsky M Marshall KA Phillippy KH Sherman PM Holko M NCBI GEO: archive for functional genomics data sets–update Nucleic acids Res 2013 41 D991 995 10.1093/nar/gks1193 23193258 PMC3531084

[B67] Li J Lenferink AE Deng Y Collins C Cui Q Purisima EO O'Connor-McCourt MD Wang E Identification of high-quality cancer prognostic markers and metastasis network modules Nat Commun 2010 1 34 20975711 10.1038/ncomms1033 PMC2972666

[B68] Montgomery SB Griffith OL Sleumer MC Bergman CM Bilenky M Pleasance ED Prychyna Y Zhang X Jones SJ ORegAnno: an open access database and curation system for literature-derived promoters, transcription factor binding sites and regulatory variation Bioinformatics 2006 22 637 640 10.1093/bioinformatics/btk027 16397004

[B69] Portales-Casamar E Kirov S Lim J Lithwick S Swanson MI Ticoll A Snoddy J Wasserman WW PAZAR: a framework for collection and dissemination of cis-regulatory sequence annotation Genome Biol 2007 8 R207 10.1186/gb-2007-8-10-r207 17916232 PMC2246282

[B70] Matys V Fricke E Geffers R Gossling E Haubrock M Hehl R Hornischer K Karas D Kel AE Kel-Margoulis OV TRANSFAC: transcriptional regulation, from patterns to profiles Nucleic acids Res 2003 31 374 378 10.1093/nar/gkg108 12520026 PMC165555

[B71] Zhao F Xuan Z Liu L Zhang MQ TRED: a Transcriptional Regulatory Element Database and a platform for in silico gene regulation studies Nucleic acids Res 2005 33 D103 107 10.1093/nar/gni105 15608156 PMC539958

[B72] Dreszer TR Karolchik D Zweig AS Hinrichs AS Raney BJ Kuhn RM Meyer LR Wong M Sloan CA Rosenbloom KR The UCSC Genome Browser database: extensions and updates 2011 Nucleic acids Res 2012 40 D918 923 10.1093/nar/gkr1055 22086951 PMC3245018

[B73] Shannon P Markiel A Ozier O Baliga NS Wang JT Ramage D Amin N Schwikowski B Ideker T Cytoscape: a software environment for integrated models of biomolecular interaction networks Genome Res 2003 13 2498 2504 10.1101/gr.1239303 14597658 PMC403769

[B74] Tusher VG Tibshirani R Chu G Significance analysis of microarrays applied to the ionizing radiation response Proc Natl Acad Sci USA 2001 98 5116 5121 10.1073/pnas.091062498 11309499 PMC33173

[B75] de Hoon MJ Imoto S Nolan J Miyano S Open source clustering software Bioinformatics 2004 20 1453 1454 10.1093/bioinformatics/bth078 14871861

[B76] Kozomara A Griffiths-Jones S miRBase: integrating microRNA annotation and deep-sequencing data Nucleic acids Res 2011 39 D152 157 10.1093/nar/gkq1027 21037258 PMC3013655

[B77] Goujon M McWilliam H Li W Valentin F Squizzato S Paern J Lopez R A new bioinformatics analysis tools framework at EMBL-EBI Nucleic acids research 2010 38 W695 699 10.1093/nar/gkq313 20439314 PMC2896090

