# Supplementary material for: Functional analysis of microRNA and transcription factor synergistic regulatory network based on identifying regulatory motifs in non-small cell lung cancer
Source: BMC Syst Biol. 2013 Nov 7;7:122. doi: 10.1186/1752-0509-7-122 (PMC3843544; doi:10.1186/1752-0509-7-122)
Supplement: Additional file 1: Table S1 — Is provided as the ratio of overlap genes and original genes after bootstrappings; Table S2 is the hub TFs and miRNAs of lung cancer synergistic regulatory network; Table S3 is the hub miRNAs and TFs of subnetwork Ito X; Table S4 is the count of motif types (subnetworks) miRNAs or TFs belong to; Table S5 shows specific functions of miRNA-TF regulatory subnetwork Ito X; Table S6 indicates target genes (E2F1 and RB1) predictive results of the miR-17 family; Table S7 is provided as differential expression analysis of the miR-17 family and RB1 by SAM; Table S8 is a list of miRNA-target relation predictive algorithms and databases used in our work. [file 1752-0509-7-122-S1.pdf]

Supplementary Table S1. The ratio of overlap genes and original genes after bootstrappings

sampling ID	overlap percentage	sampling ID	overlap percentage	sampling ID	overlap percentage	sampling ID	overlap percentage	sampling ID	overlap percentage
1	0.88	21	0.87	41	0.796	61	0.888	81	0.945
2	0.879	22	0.909	42	0.87	62	0.91	82	0.878
3	0.91	23	0.886	43	0.874	63	0.875	83	0.903
4	0.847	24	0.85	44	0.938	64	0.891	84	0.818
5	0.878	25	0.892	45	0.91	65	0.863	85	0.822
6	0.88	26	0.849	46	0.867	66	0.887	86	0.813
7	0.869	27	0.883	47	0.851	67	0.882	87	0.869
8	0.858	28	0.89	48	0.934	68	0.889	88	0.909
9	0.863	29	0.925	49	0.888	69	0.897	89	0.869
10	0.884	30	0.933	50	0.848	70	0.869	90	0.859
11	0.879	31	0.86	51	0.856	71	0.867	91	0.904
12	0.878	32	0.885	52	0.877	72	0.873	92	0.895
13	0.861	33	0.854	53	0.852	73	0.904	93	0.865
14	0.846	34	0.914	54	0.876	74	0.905	94	0.858
15	0.873	35	0.882	55	0.905	75	0.887	95	0.874
16	0.893	36	0.892	56	0.839	76	0.826	96	0.897
17	0.87	37	0.872	57	0.875	77	0.886	97	0.887
18	0.887	38	0.864	58	0.85	78	0.872	98	0.828
19	0.86	39	0.84	59	0.95	79	0.878	99	0.873
20	0.877	40	0.878	60	0.871	80	0.874	100	0.907

Supplementary Table S2. Hub TFs and miRNAs of lung cancer synergistic regulatory network

Hub TF	Degree	**Supported by**	Hub miRNA	Degree	**Supported by**
ESR1	147	PMID: 18949413	hsa-miR-590-3p	321	-
TP53	123	PMID: 12619108	hsa-miR-548c-3p	302	-
E2F3	117	PMID: 16938365	hsa-miR-570	243	-
MYC	116	PMID: 11720740	hsa-miR-340	242	-
E2F1	111	PMID: 22803943	hsa-miR-495	218	-
ETS1	109	PMID: 17785952	hsa-miR-106a *	211	PMID: 19209007
PPARA	88		hsa-miR-20a *	204	PMID: 16266980
STAT1	81		hsa-miR-106b *	203	-
SMAD7	80		hsa-miR-20b *	201	-
ETS2	71	PMID: 21922129	hsa-miR-944	200	-
			hsa-miR-93	199	-
			hsa-miR-548d-3p	196	-
			hsa-miR-545	196	-

* : belongs to miR-17 family;

Supported by : in which articls the TF or miRNA was experimental verificated to related with lung cancerdevelopment and progression

Supplementary Table S3. Hub miRNAs and TFs of subnetwork Ⅰ to Ⅹ.

Subnetwork	Hub TF	Degree	Hub miRNA	Degree
Ⅰ	E2F1	65	hsa-miR-106a	12
Ⅱ	ESR1	433	hsa-miR-590-3p	42
			hsa-miR-548d-3p	39
			hsa-miR-548c-3p	33
			hsa-miR-27b	30
			hsa-miR-302a	30
			hsa-miR-302d	30
			hsa-miR-302b	30
			hsa-miR-302c	30
			hsa-miR-152	30
			hsa-miR-495	28
			hsa-miR-421	28
Ⅲ	MYC	195	hsa-miR-106a	37
	TP53	55	hsa-miR-16	25
			hsa-miR-34a	20
Ⅳ	E2F1	1076	hsa-miR-106a	188
Ⅴ	E2F1	259	hsa-miR-17	44
Ⅵ	MYC	2096	hsa-miR-548c-3p	389
	TP53	1831	hsa-miR-340	355
	HIF1A	1804	hsa-miR-590-3p	344
	TFAP2A	1759	hsa-miR-570	323
	SP1	1506	hsa-miR-548d-3p	310
	JUN	1192	hsa-miR-944	306
	E2F4	1172	hsa-miR-548a-3p	298
	STAT1	1142	hsa-miR-374a	293
			hsa-miR-494	277
			hsa-miR-298	274
			hsa-miR-579	265
			hsa-miR-374b	265
			hsa-miR-449b	260
Ⅶ	E2F3	14904	hsa-miR-548c-3p	3447
	ESR1	14040	hsa-miR-590-3p	3357
			hsa-miR-570	2090
			hsa-miR-340	2081
			hsa-miR-495	2052
			hsa-miR-548d-3p	1996
			hsa-miR-548a-5p	1635
			hsa-miR-656	1570
			hsa-miR-20b	1520
			hsa-miR-545	1485
			hsa-miR-548d-5p	1463

			hsa-miR-548c-5p	1462
Ⅷ	MYC	2197	hsa-miR-129-5p	831
	POU2F1	1572	hsa-miR-429	751
	E2F1	1221	hsa-miR-152	676
			hsa-miR-301b	674
			hsa-miR-16	663
Ⅸ	ESR1	2717	hsa-miR-300	196
	ETS1	2506	hsa-miR-152	191
			hsa-miR-940	182
			hsa-miR-330-3p	173
			hsa-miR-381	162
			hsa-miR-150	162
			hsa-miR-380	157
			hsa-miR-421	156
			hsa-miR-642	149
			hsa-miR-19a	149
			hsa-miR-548d-3p	144
			hsa-miR-338	143
Ⅹ	MYC	1357	hsa-miR-16	174
	TP53	561	hsa-miR-92a	173
	E2F1	554	hsa-miR-34c-5p	167
			hsa-miR-34a	165
			hsa-miR-106a	151

Supplementary Table S4.1 The count of motif types (subnetworks) miRNAs belong to.

miRNA	*	miRNA	*	miRNA	*	miRNA	*
miR-381	10	miR-200b	6	miR-297	4	miR-619	4
miR-106a	10	miR-200c	6	miR-488	4	miR-30b	4
miR-20a	10	miR-330-3p	6	miR-181b	4	miR-503	4
miR-17	10	miR-940	6	miR-181a	4	miR-588	4
miR-19b	10	miR-657	6	miR-593	4	miR-30d	4
miR-21	10	miR-421	6	miR-450b-5p	4	miR-30a	4
miR-221	10	miR-511	6	miR-892a	4	miR-767-5p	4
miR-219-5p	9	miR-101	6	miR-494	4	miR-595	4
miR-16	9	miR-608	6	miR-377	4	miR-30c	4
miR-106b	7	miR-1254	6	miR-1265	4	miR-30e	4
miR-20b	7	miR-646	6	miR-507	4	miR-188-5p	4
miR-222	7	miR-153	6	miR-571	4	miR-519b-5p	4
miR-338-3p	7	miR-590-5p	6	miR-181c	4	miR-519c-5p	4
miR-484	7	miR-328	6	miR-577	4	miR-548d-5p	4
miR-429	7	miR-633	6	miR-575	4	miR-559	4
miR-590-3p	7	miR-200a	6	miR-548a-5p	4	miR-301a	4

**miR-647**	7	**miR-1291**	6	**miR-340**	4	**miR-548c-5p**	4
**miR-92a**	7	**miR-142-5p**	6	**miR-150**	4	**miR-133a**	4
**miR-199a-5p**	7	**miR-34c-5p**	6	**miR-148b**	4	**miR-133b**	4
**miR-203**	7	**miR-137**	6	**miR-148a**	4	**miR-370**	4
**miR-300**	7	**miR-208b**	6	**miR-205**	4	**miR-320a**	4
**miR-338-5p**	7	**miR-134**	5	**miR-545**	4	**miR-10b**	4
**miR-433**	7	**miR-1279**	5	**miR-149**	4	**miR-320b**	4
**miR-302a**	7	**miR-489**	4	**miR-103**	4	**miR-320c**	4
**miR-302d**	7	**miR-515-5p**	4	**miR-454**	4	**miR-320d**	4
**miR-302b**	7	**miR-548c-3p**	4	**miR-512-3p**	4	**miR-509-3-5p**	4
**miR-302c**	7	**miR-369-3p**	4	**miR-204**	4	**miR-509-5p**	4
**miR-372**	7	**miR-625**	4	**miR-211**	4	**miR-876-5p**	4
**miR-519d**	7	**miR-495**	4	**miR-767-3p**	4	**miR-605**	4
**miR-199a-3p**	7	**miR-512-5p**	4	**miR-603**	4	**miR-548l**	4
**miR-568**	7	**miR-944**	4	**miR-513a-3p**	4	**miR-449b**	4
**miR-9**	7	**miR-656**	4	**miR-548n**	4	**miR-624**	4
**miR-199b-3p**	7	**miR-544**	4	**miR-561**	4	**miR-33a**	4
**miR-129-5p**	7	**miR-524-5p**	4	**miR-510**	4	**miR-33b**	4
**miR-329**	7	**miR-570**	4	**miR-15b**	4	**miR-648**	4
**miR-548a-3p**	7	**miR-520d-5p**	4	**miR-576-3p**	4	**miR-212**	4
**miR-152**	7	**miR-548d-3p**	4	**miR-522**	4	**miR-194**	4
**miR-18b**	7	**let-7d**	4	**miR-424**	4	**miR-591**	4
**miR-107**	7	**miR-650**	4	**miR-374b**	4	**miR-935**	4
**miR-145**	7	**miR-493**	4	**miR-96**	4	**miR-618**	3
**miR-18a**	7	**miR-92b**	4	**miR-374a**	4	**miR-628-5p**	3
**miR-223**	7	**miR-520h**	4	**miR-491-3p**	4	**miR-566**	3
**miR-671-5p**	7	**miR-766**	4	**miR-497**	4	**miR-539**	3
**miR-195**	7	**miR-520g**	4	**miR-573**	4	**miR-1226**	3
**miR-19a**	7	**miR-622**	4	**miR-218**	4	**miR-499-3p**	3
**miR-15a**	7	**miR-27a**	4	**miR-217**	4	**miR-542-5p**	3
**miR-558**	7	**miR-27b**	4	**miR-130b**	4	**miR-604**	3
**miR-34a**	7	**miR-144**	4	**miR-409-3p**	4	**miR-32**	3
**miR-301b**	7	**miR-380**	4	**miR-130a**	4	**miR-128**	3
**miR-26a**	7	**let-7i**	4	**miR-26b**	4	**miR-548k**	3
**miR-423-5p**	7	**let-7e**	4	**miR-410**	4	**miR-1237**	3
**miR-634**	7	**let-7c**	4	**miR-641**	4	**miR-651**	3
**miR-361-5p**	7	**let-7b**	4	**miR-543**	4	**miR-708**	3
**miR-141**	7	**miR-298**	4	**miR-578**	4	**miR-1184**	3
**miR-448**	7	**miR-520b**	4	**miR-581**	4	**miR-1183**	3
**miR-548b-5p**	7	**miR-520e**	4	**miR-659**	4	**miR-621**	3
**miR-582-5p**	7	**miR-520a-3p**	4	**miR-542-3p**	4	**miR-208a**	3
**miR-299-5p**	7	**miR-583**	4	**miR-642**	4	**miR-376a_star**	3
**miR-25**	7	**miR-519a**	4	**miR-518a-5p**	4	**miR-30e-3p**	3
**miR-363**	7	**miR-519c-3p**	4	**miR-7**	4	**miR-34c**	2

miR-557	7	miR-519b-3p	4	miR-182	4	miR-375	1
miR-132	7	miR-579	4	miR-31	4	miR-183	1
miR-93	6	miR-888	4	miR-527	4	miR-191_star	1

Supplementary Table S4.2 The count of motif types (subnetworks) TFs belong to.

TF	*	TF	*	TF	*	TF	*
STAT1	10	PAX5	4	HOXA10	1	NFAT1	1
E2F1	10	STAT3	4	ZFP161	1	GATA6	1
ESR1	10	FOS	4	PITX3	1	HOXC11	1
E2F3	8	NFIC	4	ETV4	1	R1	1
TP53	7	CEBPB	4	RARA	1	NFAT5	1
JUNB	6	HNF1A	4	VDR	1	K3	1
ETS1	4	MEF2A	4	RARG	1	NFAT	1
FLI1	4	CREB1	4	PEG10	1	EGR4	1
PGR	4	ATF2	4	NFIA	1	CBFB	1
RELA	4	STAT5B	4	HIF1	1	CTNNBL1	1
AHR	4	NFE2L1	4	M2	1	GAS	1
PPARG	4	ZEB1	3	MEF3	1	KLF16	1
SMAD7	4	BACH1	3	HLF	1	SMAD2	1
WT1	4	FOXO3	3	AR	1	PAX8	1
PPARA	4	HMGA1	3	GATA4	1	PKNOX1	1
ETS2	4	FOSL1	3	GABPA	1	PBX2	1
BRCA1	4	JUND	3	SCRT1	1	ATF4	1
MYBL2	4	NR2F1	3	USF1	1	ZNF76	1
ARNT	4	NR2F2	3	USF2	1	ZNF143	1
XRCC5	4	NFYA	3	PHOX2A	1	BRF3	1
NFE2L2	4	NFYB	3	SNAI2	1	BRF4	1
MAFG	4	HSF1	3	HOXA5	1	RUNX2	1
MYC	4	HSF2	3	ERG	1	MXD1	1
POU2F1	4	EP300	3	MTF1	1	NAT10	1
EGR1	4	TOPORS	3	ZNF195	1	TBPL1	1
CEBPA	4	POU5F1	3	PPARD	1	TFDP1	1
TFAP2A	4	XRCC6	3	CEBPD	1	DP1	1
HIF1A	4	NFYC	3	SP2	1	NR5A2	1
NR3C1	4	SREBF1	3	HOXD9	1	SF1	1
E2F4	4	NR2E1	3	ZFHX1B	1	NFKB2	1
SP1	4	TLX1	3	HOXA7	1	THRB	1
SOX2	4	TP53BP1	2	MZF1	1	THRA	1
SPI1	4	RPA1	2	NR1I2	1	NEUROD1	1
JUN	4	BRCA2	2	ATF1	1	IL17RD	1
NFKB1	4	FUBP1	2	TFAP2B	1	TFCP2	1
HNF4A	4	ESR2	1	SMAD4	1	PAX3	1
STAT5A	4	RARB	1	OTX1	1	LHX2	1
LEF1	4	HNF4G	1	ATF3	1	DLX3	1
MYB	4	SP3	1	MBD2	1	ETV5	1

RFX1	4	FOXA2	1	ZNF148	1	GABPB2	1
STAT6	4	FOXA1	1	NR2F6	1	ZNF350	1
POU3F2	4	LOC100130009	1	NFAT2	1		

*: The number of motif types (subnetworks) miRNAs or TFs belong to.

Supplementary Table S5. Specific functions of miRNA-TF regulatory subnetwork Ⅰ to Ⅹ.

Table S5.1 Specific functions of Subnetwork Ⅰ

DNA replication

GO:0006260	DNA replication

Table S5.2 Specific functions of Subnetwork Ⅱ

Regulation of RNA/protein metabolic process

GO:0051252	regulation of RNA metabolic process
GO:0032270	positive regulation of cellular protein metabolic process
GO:0051247	positive regulation of protein metabolic process
GO:0045937	positive regulation of phosphate metabolic process

Regulation of protein modification

GO:0001934	positive regulation of protein phosphorylation
GO:0031401	positive regulation of protein modification process
GO:0042327	positive regulation of phosphorylation

Regulation of gene expression and DNA transcription

GO:0010629	negative regulation of gene expression
GO:0045892	negative regulation of transcription,DNA-dependent

Regulation of kinase activity

GO:0033674	positive regulation of kinase activity
GO:0045737	positive regulation of cyclin-dependent protein kinase activity
GO:0045860	positive regulation of protein kinase activity
GO:0051347	positive regulation of transferase activity
GO:0071902	positive regulation of protein serine/threonine kinase activity
GO:0000079	regulation of cyclin-dependent protein kinase activity

Response to UV-A

GO:0070141	response to UV-A

Regulation of cell migration

GO:0030334	regulation of cell migration
GO:2000147	positive regulation of cell motility

Regulation of programmed cell death

GO:0043068	positive regulation of programmed cell death
GO:0012502	induction of programmed cell death
GO:0006916	anti-apoptosis
GO:0006917	induction of apoptosis

Regulation or activation of Lymphocyte or T cell

GO:0031295	T cell costimulation
GO:0050863	regulation of T cell activation
GO:0050870	positive regulation of T cell activation

GO:0051249	regulation of lymphocyte activation
GO:0051251	positive regulation of lymphocyte activation

Table S5.3 Specific functions of Subnetwork Ⅲ

Regulation of programmed cell death	
GO:0043068	positive regulation of programmed cell death
GO:0012502	induction of programmed cell death
GO:0006917	induction of apoptosis
Regulation of RNA/protein metabolic process	
GO:0051252	regulation of RNA metabolic process
GO:0051247	positive regulation of protein metabolic process
GO:0016070	RNA metabolic process
GO:0032269	negative regulation of cellular protein metabolic process
GO:0051248	negative regulation of protein metabolic process
GO:0042176	regulation of protein catabolic process
Regulation of transferase activity	
GO:0051348	negative regulation of transferase activity
GO:0033673	negative regulation of kinase activity
GO:0006469	negative regulation of protein kinase activity
GO:0033674	positive regulation of kinase activity
GO:0045860	positive regulation of protein kinase activity
GO:0051347	positive regulation of transferase activity
GO:0071901	negative regulation of protein serine/threonine kinase activity
Immune reponse	
GO:0002253	activation of immune response
GO:0002757	immune response-activating signal transduction
Regulation of gene expression and DNA transcrition	
GO:0010629	negative regulation of gene expression
GO:0045892	negative regulation of transcription, DNA-dependent

Table S5.4 Specific functions of Subnetwork Ⅳ

Inositol lipid-mediated signaling	
GO:0048015	phosphatidylinositol-mediated signaling
GO:0048017	inositol lipid-mediated signaling
Regulation of protein kinase/transferase/ hydrolase activity	
GO:0006469	negative regulation of protein kinase activity
GO:0033673	negative regulation of kinase activity
GO:0045860	positive regulation of protein kinase activity
GO:0051348	negative regulation of transferase activity
GO:0033674	positive regulation of kinase activity
GO:0051347	positive regulation of transferase activity
GO:0032147	activation of protein kinase activity
GO:0043405	activation of MAP kinase activity
GO:0051345	positive regulation of hydrolase activity

GO:0061097 regulation of protein tyrosine kinase activity
GO:0071901 negative regulation of protein serine/threonine kinase activity
GO:0071902 positive regulation of protein serine/threonine kinase activity

Receptor signaling pathway

GO:0007173 epidermal growth factor receptor signaling pathway
GO:0007179 transforming growth factor beta receptor signaling pathway
GO:0008543 fibroblast growth factor receptor signaling pathway
GO:0002221 pattern recognition receptor signaling pathway
GO:0002429 immune response-activating cell surface receptor signaling pathway
GO:0002755 MyD88-dependent toll-like receptor signaling pathway
GO:0002768 immune response-regulating cell surface receptor signaling pathway
GO:0017015 regulation of transforming growth factor beta receptor signaling pathway
GO:0060070 canonical Wnt receptor signaling pathway

Kinase (signaling) cascade

GO:0007243 intracellular protein kinase cascade
GO:0000165 MAPKKK cascade
GO:0043408 regulation of MAPKKK cascade
GO:0010740 positive regulation of intracellular protein kinase cascade
GO:0010741 negative regulation of intracellular protein kinase cascade
GO:0043409 negative regulation of MAPKKK cascade
GO:0043410 positive regulation of MAPKKK cascade
GO:0007254 JNK cascade
GO:0046328 regulation of JNK cascade
GO:0046330 positive regulation of JNK cascade
GO:0070302 regulation of stress-activated protein kinase signaling cascade
GO:0070304 positive regulation of stress-activated protein kinase signaling cascade

Induction of appotosis or programmed cell death

GO:0006917 induction of apoptosis
GO:0008624 induction of apoptosis by extracellular signals
GO:0012502 induction of programmed cell death
GO:0043068 positive regulation of programmed cell death

Regulation of phosphorylation

GO:0042327 positive regulation of phosphorylation
GO:0001934 positive regulation of protein phosphorylation
GO:0018108 peptidyl-tyrosine phosphorylation
GO:0001933 negative regulation of protein phosphorylation
GO:0042326 negative regulation of phosphorylation
GO:0006470 protein dephosphorylation

Regulation of protein/phosphate catabolic process

GO:0042176 regulation of protein catabolic process
GO:0045732 positive regulation of protein catabolic process
GO:0045936 negative regulation of phosphate metabolic process
GO:0045937 positive regulation of phosphate metabolic process

Heart development

GO:0007507	heart development
Immune response	
GO:0002757	immune response-activating signal transduction
GO:0002758	innate immune response-activating signal transduction
GO:0045089	positive regulation of innate immune response
Neuron differentiation and development	
GO:0030182	neuron differentiation
GO:0048666	neuron development
Response to insulin stimulus	
GO:0032868	response to insulin stimulus
GO:0032869	cellular response to insulin stimulus

Table S5.5 Specific functions of Subnetwork V

Anti-apoptosis	
GO:0006916	anti-apoptosis
GO:0006917	induction of apoptosis
GO:0012502	induction of programmed cell death
Regulation of transcription and gene expression	
GO:0000122	negative regulation of transcription from RNA polymerase II promoter
GO:0045944	positive regulation of transcription from RNA polymerase II promoter
GO:0000083	regulation of transcription involved in G1/S phase of mitotic cell cycle
GO:0010628	positive regulation of gene expression
DNA replication	
GO:0006260	DNA replication
GO:0006261	DNA-dependent DNA replication
GO:0006271	DNA strand elongation involved in DNA replication
GO:0022616	DNA strand elongation
DNA damage repair	
GO:0006284	base-excision repair
GO:0006298	mismatch repair
GO:0006302	double-strand break repair
GO:0000077	DNA damage checkpoint
GO:0042770	signal transduction in response to DNA damage
GO:0030330	DNA damage response, signal transduction by p53 class mediator
Telomere mainteance and organization	
GO:0010833	telomere maintenance via telomere lengthening
GO:0000722	telomere maintenance via recombination
GO:0000723	telomere maintenance
GO:0032200	telomere organization
GO:0032201	telomere maintenance via semi-conservative replication
Amino acid/purine nucleotide metabolic process	
GO:0006520	cellular amino acid metabolic process
GO:0006575	cellular modified amino acid metabolic process
GO:0006163	purine nucleotide metabolic process

GO:0006195	purine nucleotide catabolic process
GO:0009166	nucleotide catabolic process
GO:0045937	positive regulation of phosphate metabolic process
GO:0032270	positive regulation of cellular protein metabolic process
Regulation of hydrolase/protein kinase activity	
GO:0051345	positive regulation of hydrolase activity
GO:0000079	regulation of cyclin-dependent protein kinase activity
GO:0043405	regulation of MAP kinase activity
GO:0033674	positive regulation of kinase activity
GO:0045737	positive regulation of cyclin-dependent protein kinase activity
GO:0045860	positive regulation of protein kinase activity
GO:0050999	regulation of nitric-oxide synthase activity
GO:0051347	positive regulation of transferase activity
GO:0071902	positive regulation of protein serine/threonine kinase activity
GO:0002696	positive regulation of leukocyte activation
Regulation of cell migration	
GO:0030334	regulation of cell migration
GO:2000147	positive regulation of cell motility
Response to insulin stimulus	
GO:0032868	response to insulin stimulus
GO:0032869	cellular response to insulin stimulus

Table S5.6 Specific functions of Subnetwork Ⅵ

Regulation of RNA/protein metabolic process	
GO:0051252	regulation of RNA metabolic process
GO:0045937	positive regulation of phosphate metabolic process
GO:0032270	positive regulation of cellular protein metabolic process
GO:0042176	regulation of protein catabolic process
GO:0051247	positive regulation of protein metabolic process
Regulation of programmed cell death or apoptosis	
GO:0043068	positive regulation of programmed cell death
GO:0006917	induction of apoptosis
GO:0012502	induction of programmed cell death
GO:0006916	anti-apoptosis
GO:0090068	positive regulation of cell cycle process
Regulation of cell migration	
GO:0030334	regulation of cell migration
GO:2000147	positive regulation of cell motility
GO:0030335	positive regulation of cell migration
Regulation of hydrolase/kinase activity	
GO:0051345	positive regulation of hydrolase activity
GO:0033674	positive regulation of kinase activity
GO:0051347	positive regulation of transferase activity
GO:0045860	positive regulation of protein kinase activity

GO:0000079	regulation of cyclin-dependent protein kinase activity
GO:0071902	positive regulation of protein serine/threonine kinase activity
GO:0051348	negative regulation of transferase activity
GO:0045737	positive regulation of cyclin-dependent protein kinase activity
GO:0033673	negative regulation of kinase activity
GO:0006469	negative regulation of protein kinase activity
Neuron generation,differentiation and development and cell morphogenesis	
GO:0048699	generation of neurons
GO:0030182	neuron differentiation
GO:0048666	neuron development
GO:0007409	axonogenesis
GO:0048667	cell morphogenesis involved in neuron differentiation
GO:0048812	neuron projection morphogenesis
GO:0007411	axon guidance
GO:0000904	cell morphogenesis involved in differentiation
GO:0048858	cell projection morphogenesis
GO:0031175	neuron projection development
Protein modification	
GO:0031401	positive regulation of protein modification process
GO:0001934	positive regulation of protein phosphorylation
GO:0018193	peptidyl-amino acid modification

Table S5.7 Specific functions of Subnetwork Ⅶ

Fibroblast growth factor signaling pathway and cellular response	
GO:0008543	fibroblast growth factor receptor signaling pathway
GO:0044344	cellular response to fibroblast growth factor stimulus
Inositol lipid-mediated signaling	
GO:0048017	inositol lipid-mediated signaling
Response to insulin stimulus	
GO:0032869	cellular response to insulin stimulus
GO:0032868	response to insulin stimulus
Regulation of MAPK cascade	
GO:0007243	intracellular protein kinase cascade
GO:0010741	negative regulation of intracellular protein kinase cascade
GO:0043408	regulation of MAPKKK cascade
GO:0043410	positive regulation of MAPKKK cascade
GO:0043409	negative regulation of MAPKKK cascade
GO:0051403	stress-activated MAPK cascade
GO:0000165	MAPKKK cascade
Regulation of receptor signaling pathway	
GO:0007178	transmembrane receptor protein serine/threonine kinase signaling pathway
GO:0007173	epidermal growth factor receptor signaling pathway
GO:0008286	insulin receptor signaling pathway
GO:0060828	regulation of canonical Wnt receptor signaling pathway

GO:0007179	transforming growth factor beta receptor signaling pathway
GO:0060070	canonical Wnt receptor signaling pathway
GO:0030177	positive regulation of Wnt receptor signaling pathway
GO:0090263	positive regulation of canonical Wnt receptor signaling pathway
Regulation of cell migration	
GO:0030334	regulation of cell migration
GO:0010594	regulation of endothelial cell migration
Regulation of DNA replication and metabolic	
GO:0006275	regulation of DNA replication
GO:0051054	positive regulation of DNA metabolic process
Regulation of phosphorylation	
GO:0045937	positive regulation of phosphate metabolic process
GO:0042327	positive regulation of phosphorylation
GO:0001934	positive regulation of protein phosphorylation
GO:0018108	peptidyl-tyrosine phosphorylation
GO:0042326	negative regulation of phosphorylation
GO:0001933	negative regulation of protein phosphorylation
Regulation of enzymatic activity	
GO:0051348	negative regulation of transferase activity
GO:0000079	regulation of cyclin-dependent protein kinase activity
GO:0051347	positive regulation of transferase activity
GO:0006469	negative regulation of protein kinase activity
GO:0033674	positive regulation of kinase activity
GO:0033673	negative regulation of kinase activity
GO:0045860	positive regulation of protein kinase activity
GO:0071901	negative regulation of protein serine/threonine kinase activity
GO:0051345	positive regulation of hydrolase activity
GO:0000186	activation of MAPKK activity
GO:0043087	regulation of GTPase activity
GO:0032318	regulation of Ras GTPase activity
Regulation of meiosis	
GO:0007126	meiosis
GO:0007088	regulation of mitosis
GO:0051327	M phase of meiotic cell cycle
GO:0007131	reciprocal meiotic recombination
GO:0007127	meiosis I

Table S5.8 Specific functions of Subnetwork Ⅷ

Intracellular protein kinase cascade	
GO:0007243	intracellular protein kinase cascade
GO:0010741	negative regulation of intracellular protein kinase cascade
Growth factor receptor signaling pathway	
GO:0007173	epidermal growth factor receptor signaling pathway
GO:0008543	fibroblast growth factor receptor signaling pathway

GO:0008286	insulin receptor signaling pathway
GO:0060828	regulation of canonical Wnt receptor signaling pathway
GO:0007179	transforming growth factor beta receptor signaling pathway
GO:0050852	T cell receptor signaling pathway
Response to stimulus	
GO:0032869	cellular response to insulin stimulus
GO:0032868	response to insulin stimulus
GO:0044344	cellular response to fibroblast growth factor stimulus
GO:0071375	cellular response to peptide hormone stimulus
Regulation of phosphorylation	
GO:0001934	positive regulation of protein phosphorylation
GO:0042327	positive regulation of phosphorylation
GO:0045937	positive regulation of phosphate metabolic process
GO:0031401	positive regulation of protein modification process
GO:0018108	peptidyl-tyrosine phosphorylation
MAPKKK cascade	
GO:0000165	MAPKKK cascade
GO:0043408	regulation of MAPKKK cascade
Regulation of cell migration	
GO:0030334	regulation of cell migration
GO:0010594	regulation of endothelial cell migration
GO:2000147	positive regulation of cell motility
GO:0030335	positive regulation of cell migration
Regulation of kinase activity	
GO:0033674	positive regulation of kinase activity
GO:0051347	positive regulation of transferase activity
GO:0051348	negative regulation of transferase activity
GO:0000079	regulation of cyclin-dependent protein kinase activity
GO:0045860	positive regulation of protein kinase activity
GO:0006469	negative regulation of protein kinase activity
GO:0033673	negative regulation of kinase activity
Protein stabilization	
GO:0031647	regulation of protein stability
GO:0050821	protein stabilization
Immune response	
GO:0002253	activation of immune response
GO:0002429	immune response-activating cell surface receptor signaling pathway
GO:0031295	T cell costimulation
GO:0002521	leukocyte differentiation

Table S5.9 Specific functions of Subnetwork Ⅸ

Regulation of protein metabolic process	
GO:0051247	positive regulation of protein metabolic process
GO:0032270	positive regulation of cellular protein metabolic process

Regulation of gene expression and transcription factor activity

GO:0010628	positive regulation of gene expression
GO:0045944	positive regulation of transcription from RNA polymerase II promoter
GO:0045892	negative regulation of transcription, DNA-dependent
GO:0010629	negative regulation of gene expression
GO:0051091	positive regulation of sequence-specific DNA binding transcription factor activity
GO:0051090	regulation of sequence-specific DNA binding transcription factor activity

Regulation of kinase activity

GO:0045860	positive regulation of protein kinase activity
GO:0033674	positive regulation of kinase activity
GO:0051347	positive regulation of transferase activity

Apoptosis or programmed cell death

GO:0006917	induction of apoptosis
GO:0012502	induction of programmed cell death
GO:0006916	anti-apoptosis
GO:0043068	positive regulation of programmed cell death
GO:0043281	regulation of cysteine-type endopeptidase activity involved in apoptotic process
GO:0043280	positive regulation of cysteine-type endopeptidase activity involved in apoptotic process
GO:0008624	induction of apoptosis by extracellular signals

Regulation of protein modification process

GO:0031401	positive regulation of protein modification process
GO:0042327	positive regulation of phosphorylation
GO:0001934	positive regulation of protein phosphorylation

Regulation of cell migration

GO:0030334	regulation of cell migration
GO:2000147	positive regulation of cell motility
GO:0030335	positive regulation of cell migration
GO:0010594	regulation of endothelial cell migration
GO:0010595	positive regulation of endothelial cell migration

Enzyme activity

GO:0051345	positive regulation of hydrolase activity
GO:0000079	regulation of cyclin-dependent protein kinase activity
GO:0071902	positive regulation of protein serine/threonine kinase activity
GO:0052547	regulation of peptidase activity
GO:0052548	regulation of endopeptidase activity
GO:0006919	activation of caspase activity
GO:0010950	positive regulation of endopeptidase activity
GO:0010952	positive regulation of peptidase activity

Platelet activation

GO:0030168	platelet activation

Regulation of kinase cascade

GO:0010740	positive regulation of intracellular protein kinase cascade
GO:0007243	intracellular protein kinase cascade
GO:0000165	MAPKKK cascade
GO:0043123	positive regulation of I-kappaB kinase/NF-kappaB cascade
GO:0043122	regulation of I-kappaB kinase/NF-kappaB cascade
regulation of lymphocyte activation	
GO:0002521	leukocyte differentiation
GO:0051249	regulation of lymphocyte activation
GO:0002696	positive regulation of leukocyte activation
GO:0051251	positive regulation of lymphocyte activation
GO:0031295	T cell costimulation
GO:0050870	positive regulation of T cell activation
GO:0050863	regulation of T cell activation
Regulation of mitotic cell cycle	
GO:0000077	DNA damage checkpoint
GO:0006289	nucleotide-excision repair
GO:0090068	positive regulation of cell cycle process
GO:0007050	cell cycle arrest
GO:0031571	mitotic cell cycle G1/S transition DNA damage checkpoint
Regulation of Endothelia and epithelial cell proliferation	
GO:0050679	positive regulation of epithelial cell proliferation
GO:0000904	cell morphogenesis involved in differentiation
GO:0001936	regulation of endothelial cell proliferation
GO:0001938	positive regulation of endothelial cell proliferation
Response to insulin and hormone stimulus	
GO:0071375	cellular response to peptide hormone stimulus
GO:0032868	response to insulin stimulus
GO:0032869	cellular response to insulin stimulus
GO:0043627	response to estrogen stimulus
Immune response	
GO:0045088	regulation of innate immune response
GO:0002757	immune response-activating signal transduction
GO:0002253	activation of immune response
Vessel development	
GO:0001944	vasculature development
GO:0001568	blood vessel development
GO:0001525	angiogenesis
GO:0048514	blood vessel morphogenesis
Telomere maintenance	
GO:0000723	telomere maintenance
GO:0032200	telomere organization
GO:0010833	telomere maintenance via telomere lengthening

Table S5. 10 Specific functions of Subnetwork Ⅹ

Regulation of protein metabolic process	
GO:0051247	positive regulation of protein metabolic process
GO:0032270	positive regulation of cellular protein metabolic process
GO:0006520	cellular amino acid metabolic process
Apoptosis and programmed cell death	
GO:0043068	positive regulation of programmed cell death
GO:0006916	anti-apoptosis
GO:0006917	induction of apoptosis
GO:0012502	induction of programmed cell death
GO:0043280	positive regulation of cysteine-type endopeptidase activity involved in apoptotic process
GO:0008629	induction of apoptosis by intracellular signals
Regulation of gene expression	
GO:0010628	positive regulation of gene expression
GO:0045944	positive regulation of transcription from RNA polymerase II promoter
GO:0000122	negative regulation of transcription from RNA polymerase II promoter
GO:0010629	negative regulation of gene expression
Regulation of phosphorylation	
GO:0031401	positive regulation of protein modification process
GO:0042327	positive regulation of phosphorylation
GO:0045937	positive regulation of phosphate metabolic process
GO:0001934	positive regulation of protein phosphorylation
Regulation of enzyme activity	
GO:0051345	positive regulation of hydrolase activity
GO:0000079	regulation of cyclin-dependent protein kinase activity
GO:0052547	regulation of peptidase activity
GO:0052548	regulation of endopeptidase activity
GO:0006919	activation of caspase activity
Mitotic cell cycle	
GO:0000077	DNA damage checkpoint
GO:0090068	positive regulation of cell cycle process
GO:0000083	regulation of transcription involved in G1/S phase of mitotic cell cycle
GO:0000080	G1 phase of mitotic cell cycle
GO:0006260	DNA replication
GO:0006284	base-excision repair
GO:0006302	double-strand break repair
GO:0006298	mismatch repair
GO:0007050	cell cycle arrest
GO:0007126	meiosis
GO:0007127	meiosis I
Telomere maintenance	
GO:0000723	telomere maintenance
GO:0032200	telomere organization
Regulation of kinase cascade	

GO:0010740	positive regulation of intracellular protein kinase cascade
GO:0007243	intracellular protein kinase cascade
GO:0000165	MAPKKK cascade
Inositol lipid-mediated signaling	
GO:0048017	inositol lipid-mediated signaling
ATP metabolic process	
GO:0046034	ATP metabolic process
GO:0006200	ATP catabolic process

Supplementary Table S6. Target genes (E2F1 and RB1) predictive results of the miR-17 family

	01_DT	02_miRSV	06_TS	07_PITA	08_mirT	09_tarM	10_miR
miR-17	128	746	0	1541	16	190	1418.5
	979	1031	0	2036.5	274	563	0
miR-20a	440	724	0	1491	39.5	235	1418.5
	1633	1101	510.5	1993	295	749	0
miR-20b	130	692	0	1496	38	197	1418.5
	981	1057	579.5	1997.5	401	749	0
miR-106a	455	746	0	1541	16	257	1418.5
	1939	1031	628.5	2036.5	274	765	0
miR-106b	441	672	0	1539	12	252	1418.5
	1637	995	0	2000	429	640	0
miR-93	128	690	0	1402	85	236	1418.5
	980	1070.5	0	1934	451	440	0

Non-zero values means the genes were predicted as target genes of according miRNAs; The results of E2F1 and RB1 were in even and odd rows respectively; The full name of predictive algorithms can be find in supplementary table S8.

Supplementary Table S7. Differential expression analysis of the miR-17 family and RB1 by SAM

	**Gene ID**	**GEO profile**	**Score(d)**	**Numerator (r)**	**Denominator(s+s0)**	**Fold Change**	**q-value(%)**
**miR-17**	42650	GSE27705	1.70452191	0.755263158	0.443093841	1.687939456	4.174595
**miR-106b**	19582	GSE27705	2.76738915	0.858947368	0.310381852	1.81371449	0
**miR-106a**	46801	GSE27705	1.76117657	0.767	0.435504318	1.701727459	4.174595
**miR-20a**	145845	GSE27705	1.73238454	0.761315789	0.408893141	1.695035853	4.174595
**miR-93**	30687	GSE27705	3.76611269	1.184526316	0.314522271	2.27288756	0
**RB1**	5925	GSE11969	2.39500027	0.110474348	0.046127071	1.079583138	3.68302739
	5925	GSE2088	1.34337294	0.561158588	0.417723606	1.475453637	5.0241278

Supplementary Table S8. List of miRNA-target relation predictive algorithms and databases used in our work.

Algorithm name	Abbr	Version	Website	Reference
DIANA-microT	01_DT	V3.0	http://diana.cslab.ece.ntua.gr/microT/	[1,2]
mirSVR	02_miRSV	2010.8	http://www.microrna.org/microrna/getDownloads.do	[3]
PicTar5	03_PT5	2007	http://pictar.mdc-berlin.de/	[4]
RNA22	04_R3/05_R5	2006	http://cbcsrv.watson.ibm.com/rna22.html	[5]
RNAhybrid	05_Rh	2004	http://bibiserv.techfak.uni-bielefeld.de/rnahybrid/	[6]
TargetScan	06_TS	5.2	http://www.targetscan.org/	[7,8]
PITA	07_PITA	version 6 (08.8)	http://genie.weizmann.ac.il/pubs/mir07/mir07_data.html	[9]
MirTarget2	08_mirT	2009.4	http://mirdb.org/miRDB/	[10,11]
TargetMiner	09_tarM	2009	http://www.isical.ac.in/~bioinfo_miu/targetminer20.htm	[12]
miRanda	10_miR	2010.8	http://www.microrna.org/microrna/getDownloads.do	[13,14]

1. Maragkakis M, Alexiou P, Papadopoulos GL, Reczko M, Dalamagas T, Giannopoulos G, Goumas G, Koukis E, Kourtis K, Simossis VA, et al. 2009 Accurate microRNA target prediction correlates with protein repression levels. *BMC Bioinformatics* **10**, 295.

2. Maragkakis M, Reczko M, Simossis VA, Alexiou P, Papadopoulos GL, Dalamagas T, Giannopoulos G, Goumas G, Koukis E, Kourtis K, et al. 2009 DIANA-microT web server: elucidating microRNA functions through target prediction. *Nucleic Acids Res* **37**, W273-276.

3. Betel D, Koppal A, Agius P, Sander C, Leslie C. 2010 Comprehensive modeling of microRNA targets predicts functional non-conserved and non-canonical sites. *Genome Biol* **11**, R90.

4. Krek A, Grun D, Poy MN, Wolf R, Rosenberg L, Epstein EJ, MacMenamin P, da Piedade I, Gunsalus KC, Stoffel M, et al. 2005 Combinatorial microRNA target predictions. *Nat Genet* **37**, 495-500.

5. Miranda KC, Huynh T, Tay Y, Ang YS, Tam WL, Thomson AM, Lim B, Rigoutsos I. 2006 A pattern-based method for the identification of MicroRNA binding sites and their corresponding heteroduplexes. *Cell* **126**, 1203-1217.

6. Rehmsmeier M, Steffen P, Hochsmann M, Giegerich R. 2004 Fast and effective prediction of microRNA/target duplexes. *RNA* **10**, 1507-1517.

7. Lewis BP, Shih IH, Jones-Rhoades MW, Bartel DP, Burge CB. 2003 Prediction of mammalian microRNA targets. *Cell* **115**, 787-798.

8. Lewis BP, Burge CB, Bartel DP. 2005 Conserved seed pairing, often flanked by adenosines, indicates that thousands of human genes are microRNA targets. *Cell* **120**, 15-20.

9. Kertesz M, Iovino N, Unnerstall U, Gaul U, Segal E. 2007 The role of site accessibility in microRNA target recognition. *Nat Genet* **39**, 1278-1284.

10. Wang X, El Naqa IM. 2008 Prediction of both conserved and nonconserved microRNA targets in animals. *Bioinformatics* **24**, 325-332.

11. Wang X. 2008 miRDB: a microRNA target prediction and functional annotation database with a wiki interface. *RNA* **14**, 1012-1017.

12. Bandyopadhyay S, Mitra R. 2009 TargetMiner: microRNA target prediction with systematic identification of tissue-specific negative examples. *Bioinformatics* **25**, 2625-2631.

13. John B, Enright AJ, Aravin A, Tuschl T, Sander C, Marks DS. 2004 Human MicroRNA targets. *PLoS Biol* **2**, e363.

14. Enright AJ, John B, Gaul U, Tuschl T, Sander C, Marks DS. 2003 MicroRNA targets in Drosophila. *Genome Biol* **5**, R1.

Supplementary Figure S1 to S10. miRNA-TF synergetic regulatory subnetwork Ⅰ to Ⅹ in order.

Figure S1. Subnetwork Ⅰ

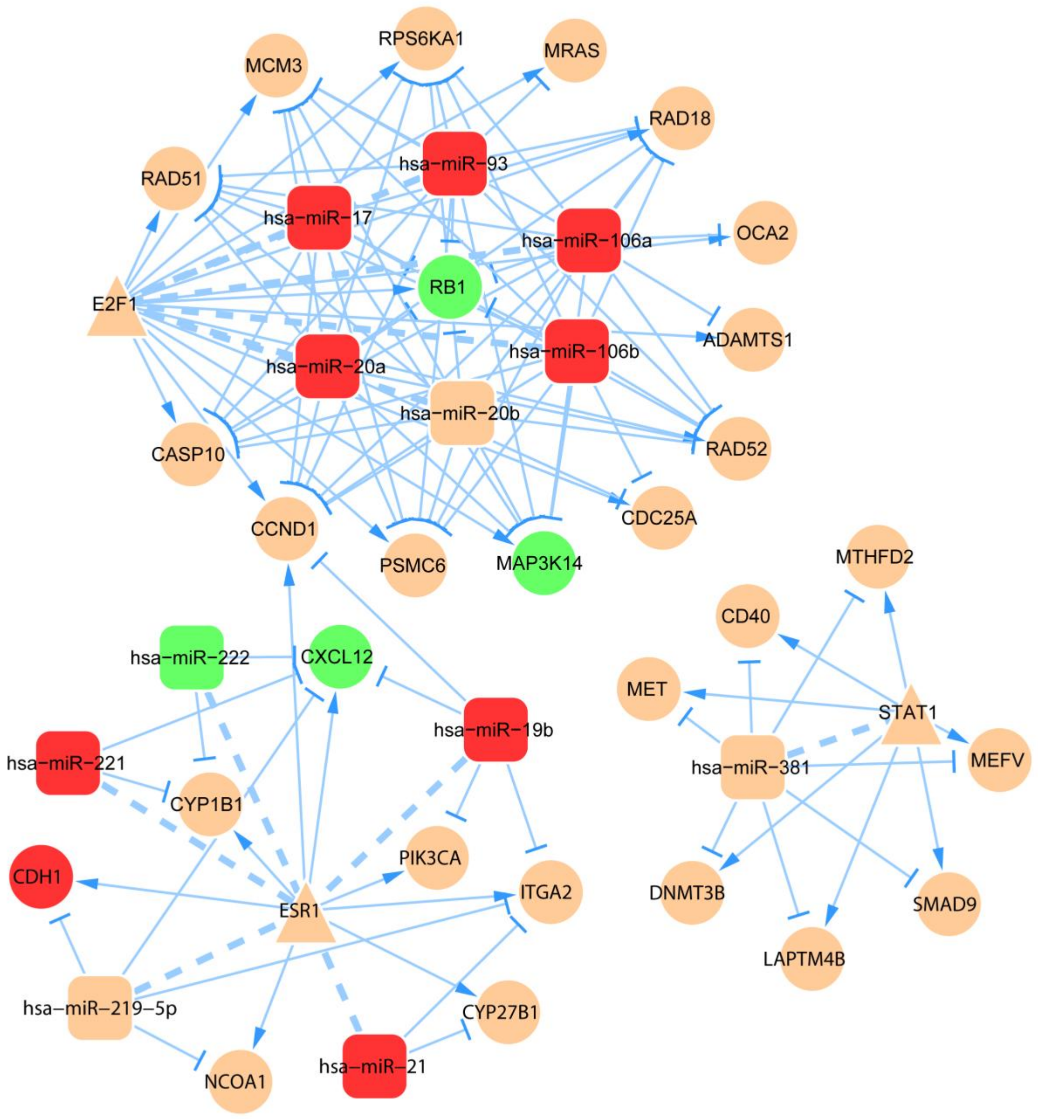

Figure S2. Subnetwork Ⅱ

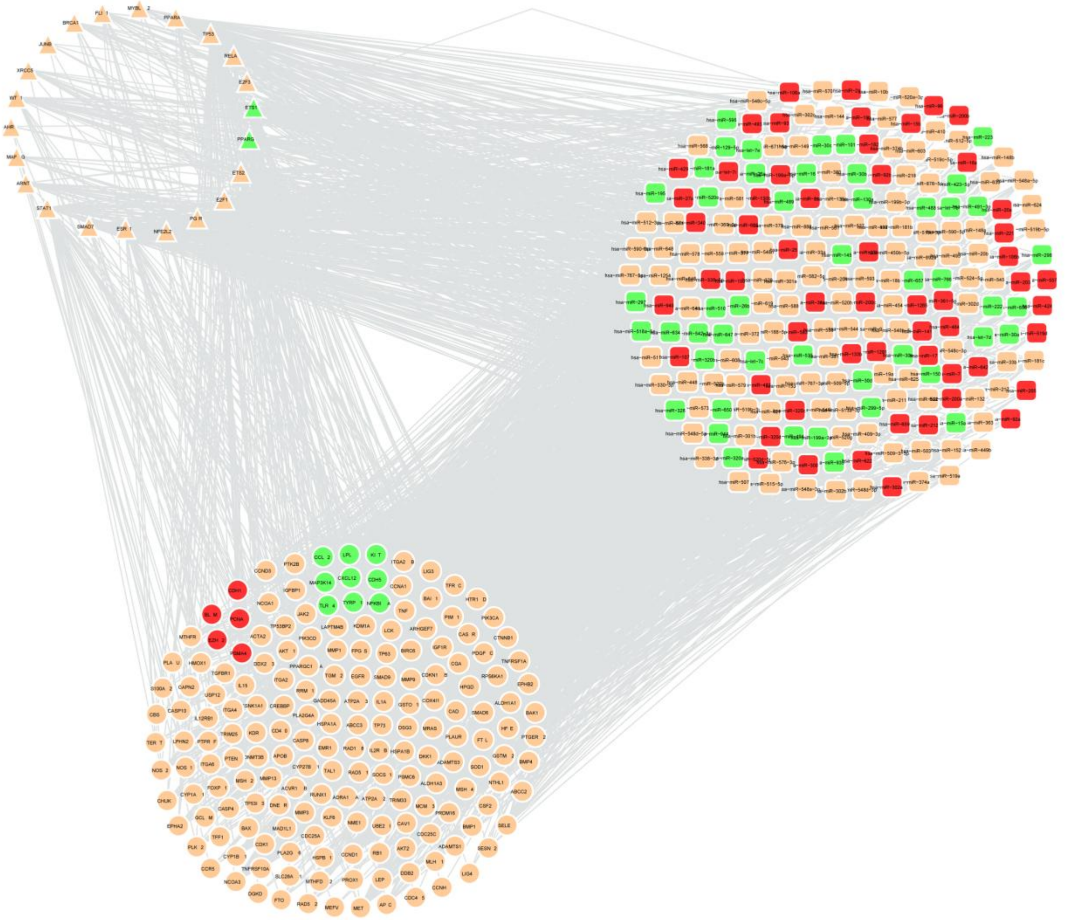

Figure S3. Subnetwork Ⅲ

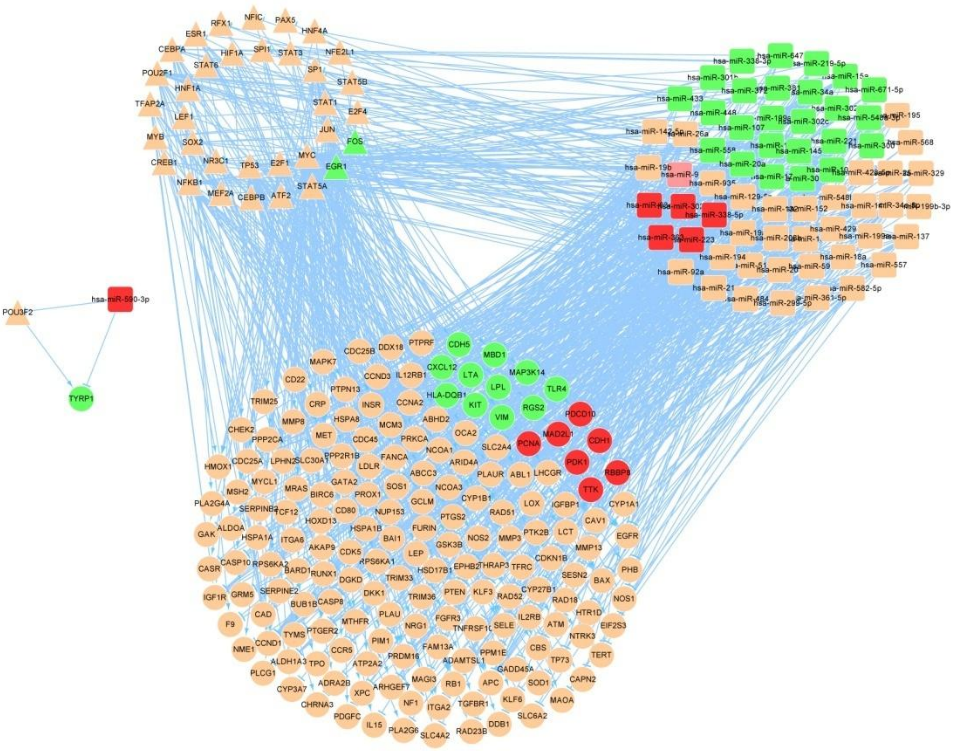

Figure S4. Subnetwork Ⅳ

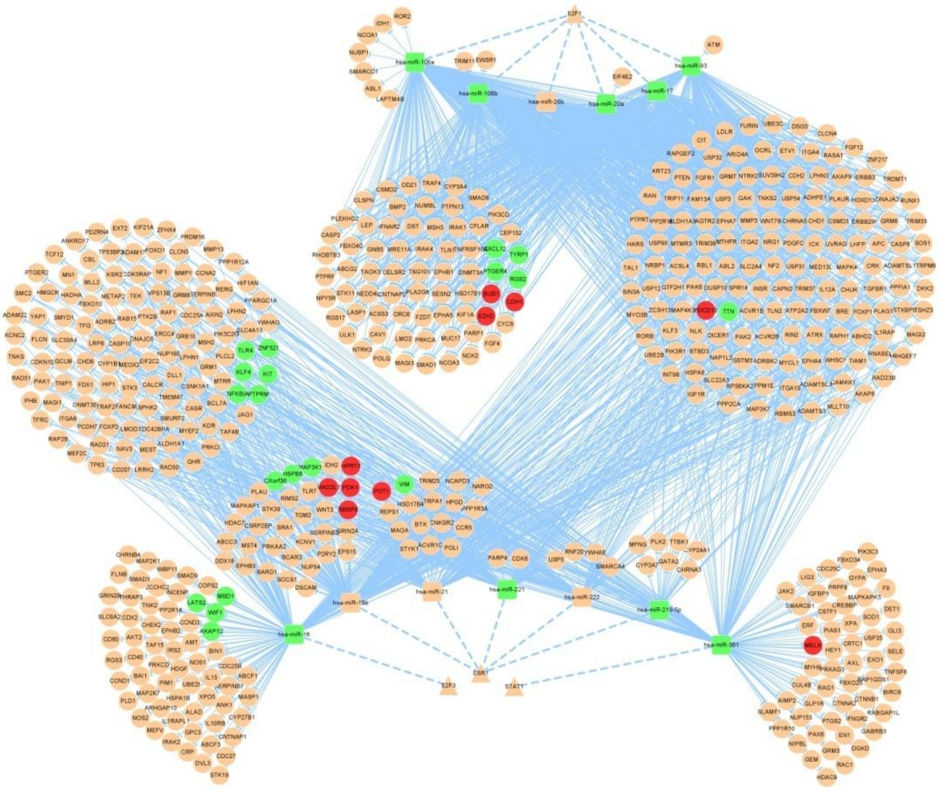

Figure S5. Subnetwork Ⅴ

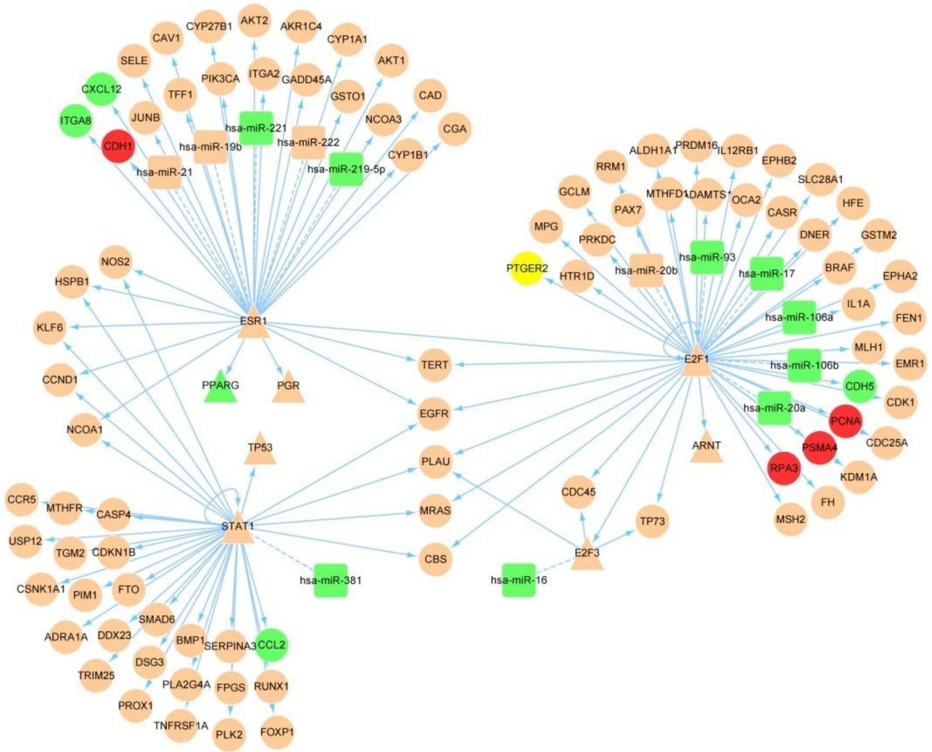

Figure S6. Subnetwork Ⅵ

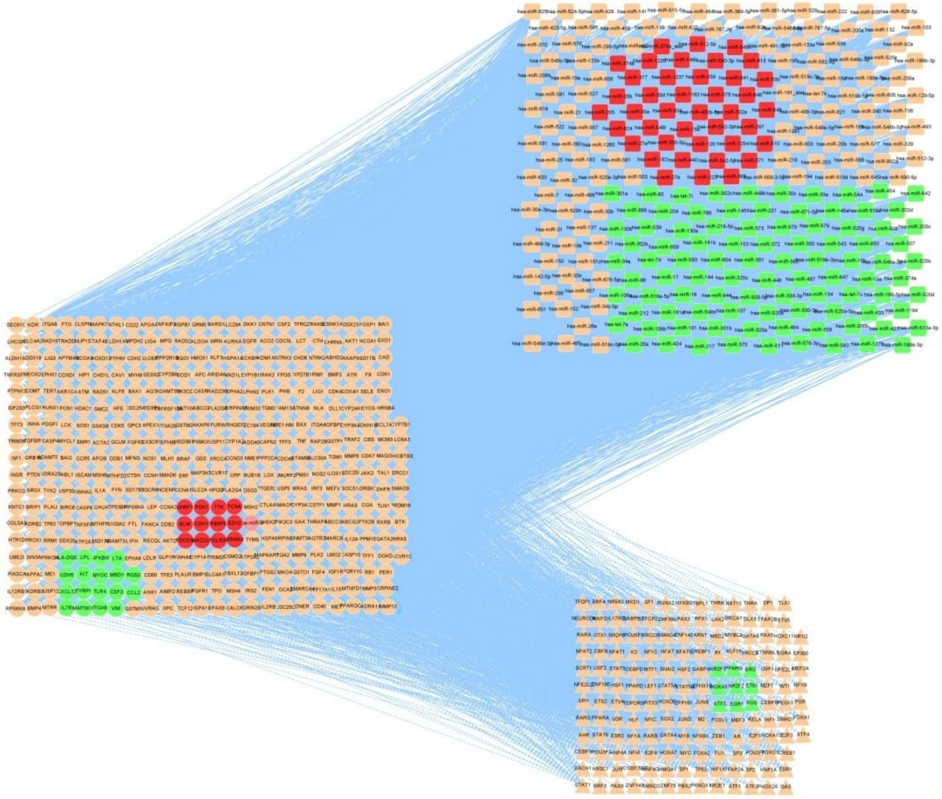

Figure S7. Subnetwork Ⅶ

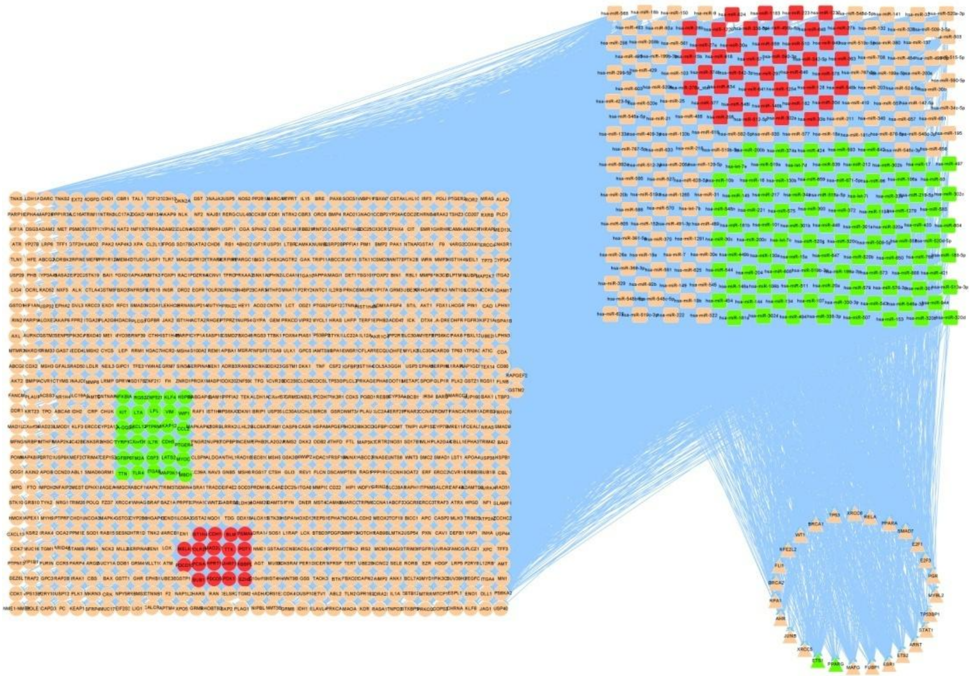

Figure S8. Subnetwork Ⅷ

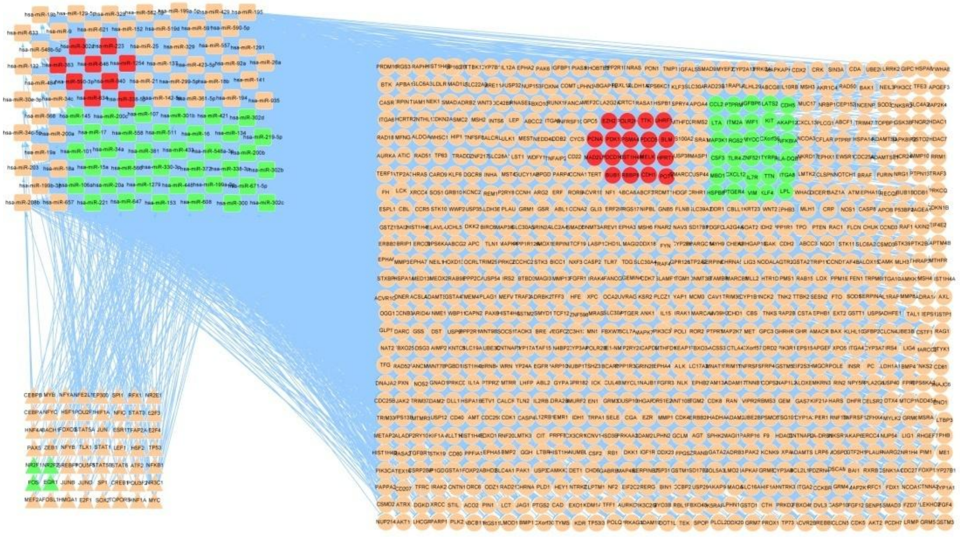

Figure S9. Subnetwork Ⅸ

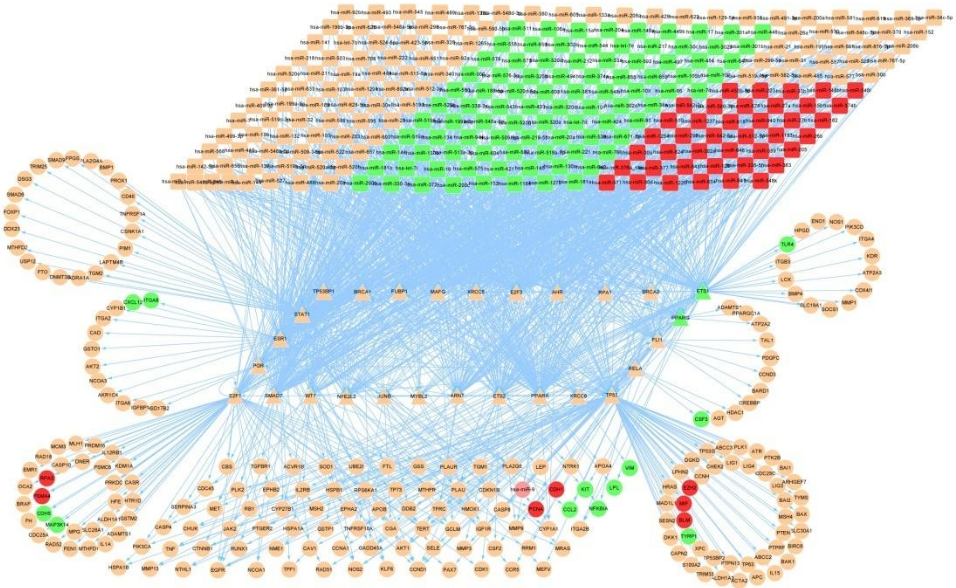

Figure S10. Subnetwork Ⅹ

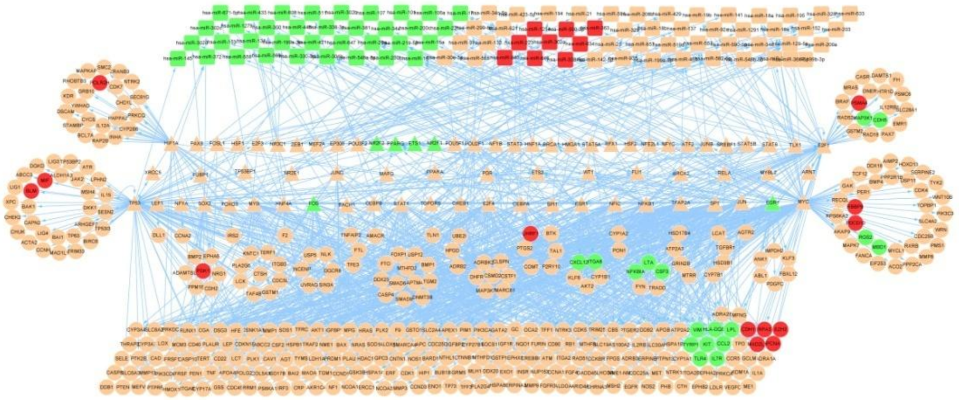

Ellipse nodes: Genes; round rectangle nodes:miRNAs; triangle nodes: TFs. Green nodes: down-regulated nodes; red nodes: up-regulated nodes. Arrow shape edge: transcriptional activation/repression; T shape dege: miRNA repression. Dash line: a feedback loop.

Figure S11. Box plots of nodes incoming degree and outgoing degree. of Gene, miRNA and TF (A) Incoming degree of miRNA, TF and gene; (B) Outgoing degree of miRNA and TF.

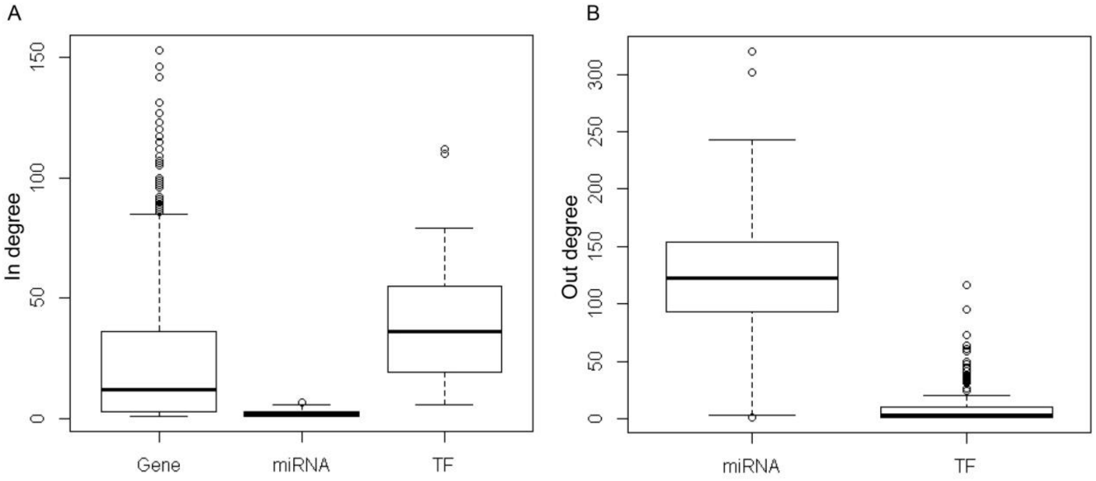

Supplementary Note. multiple sequence alignment of miR-17 family (from 3’ to 5’) and 3’-UTR region of E2F1 conserved sequences(from 5’ to 3’)

```
miR-106a          AAAAGUGCUUACAGUGCAGGUAG 23
miR-106b          UAAAGUGCUGACAGUGCAGAU-- 21
miR-20a           UAAAGUGCUUAUAGUGCAGGUAG 23
miR-17            CAAAGUGCUUACAGUGCAGGUAG 23
miR-20b           CAAAGUGCUCAUAGUGCAGGUAG 23
miR-93            CAAAGUGCUGUUCGUGCAGGUAG 23
                   ********    ****** *
```

gi|189217864|ref|NM_001100778.1| Rattus norvegicus E2F transcription factor 1 (E2f1), mRNA
gi|158517881|ref|NM_007891.4| Mus musculus E2F transcription factor 1 (E2f1), mRNA
gi|329663320|ref|NM_001206079.1| Bos taurus E2F transcription factor 1 (E2F1), mRNA
gi|168480109|ref|NM_005225.2| Homo sapiens E2F transcription factor 1 (E2F1), mRNA
gi|332858149|ref|XM_003316862.1| PREDICTED: Pan troglodytes E2F transcription factor 1 (E2F1), mRNA

```
miR-17                              ------gauggacgugacauucgugaaac----------
                                          ||| | ||||| |  |     |
gi|189217864|ref|NM_001100778.1|    ugggcucuaacagcacuuuugguguccuugcugcagggg
gi|332858149|ref|XM_003316862.1|    ggggcucuaacugcacuuucggcccuuuugcucuggggg
gi|329663320|ref|NM_001206079.1|    ggggcucuaacugcacuuuuggucuucuugcuccagggg
gi|168480109|ref|NM_005225.2|       ggggcucuaacugcacuuucggcccuuuugcucuggggg
gi|158517881|ref|NM_007891.4|       ugggcucuaacugcacuuuugguguccuugcugcagggg


miR-106a                            ------gauggacgugacauucgugaaaa----------
                                          ||| | ||||| |  |     ||
gi|189217864|ref|NM_001100778.1|    ugggcucuaacagcacuuuugguguccuugcugcagggg
gi|332858149|ref|XM_003316862.1|    ggggcucuaacugcacuuucggcccuuuugcucuggggg
gi|329663320|ref|NM_001206079.1|    ggggcucuaacugcacuuuuggucuucuugcuccagggg
gi|168480109|ref|NM_005225.2|       ggggcucuaacugcacuuucggcccuuuugcucuggggg
gi|158517881|ref|NM_007891.4|       ugggcucuaacugcacuuuugguguccuugcugcagggg


miR-106b                            ---------uagacgugacagucgugaaau---------
                                             | | ||||| |  |     |
gi|189217864|ref|NM_001100778.1|    gugggcucuaacagcacuuuugguguccuugcugcaggg
gi|332858149|ref|XM_003316862.1|    -ggggcucuaacugcacuuucggcccuuuugcucugggg
gi|329663320|ref|NM_001206079.1|    gggggcucuaacugcacuuuuggucuucuugcuccaggg
gi|168480109|ref|NM_005225.2|       -ggggcucuaacugcacuuucggcccuuuugcucugggg
gi|158517881|ref|NM_007891.4|       gugggcucuaacugcacuuuugguguccuugcugcaggg

miR-20a                             ------gauggacgugauauucgugaaau----------
                                          ||| | ||||| |  |     |
gi|189217864|ref|NM_001100778.1|    ugggcucuaacagcacuuuugguguccuugcugcagggg
gi|332858149|ref|XM_003316862.1|    ggggcucuaacugcacuuucggcccuuuugcucuggggg
gi|329663320|ref|NM_001206079.1|    ggggcucuaacugcacuuuuggucuucuugcuccagggg
gi|168480109|ref|NM_005225.2|       ggggcucuaacugcacuuucggcccuuuugcucuggggg
```

```
gi|158517881|ref|NM_007891.4|       ugggcucuaacugcacuuuuggugucccuugcugcagggg

miR-20b                             ------gauggacgugauacucgugaaac----------
                                          ||| | ||||| |  |      |
gi|189217864|ref|NM_001100778.1|    ugggcucuaacagcacuuuuggugucccuugcugcagggg
gi|332858149|ref|XM_003316862.1|    ggggcucuaacugcacuuucggcccuuuugcucuggggg
gi|329663320|ref|NM_001206079.1|    ggggcucuaacugcacuuuuggucuucuugcuccagggg
gi|168480109|ref|NM_005225.2|       ggggcucuaacugcacuuucggcccuuuugcucuggggg
gi|158517881|ref|NM_007891.4|       ugggcucuaacugcacuuuuggugucccuugcugcagggg

miR-93                              ------gauggacgugcuu-------------------
                                          ||| | ||||
gi|189217864|ref|NM_001100778.1|    ugggcucuaacagcacuuuuggugucccuugcugcagggg
gi|332858149|ref|XM_003316862.1|    ggggcucuaacugcacuuucggcccuuuugcucuggggg
gi|329663320|ref|NM_001206079.1|    ggggcucuaacugcacuuuuggucuucuugcuccagggg
gi|168480109|ref|NM_005225.2|       ggggcucuaacugcacuuucggcccuuuugcucuggggg
gi|158517881|ref|NM_007891.4|       ugggcucuaacugcacuuuuggugucccuugcugcagggg
```


1. Maragkakis M, Alexiou P, Papadopoulos GL, Reczko M, Dalamagas T, et al. (2009) Accurate microRNA target prediction correlates with protein repression levels. BMC Bioinformatics 10: 295.
2. Maragkakis M, Reczko M, Simossis VA, Alexiou P, Papadopoulos GL, et al. (2009) DIANA-microT web server: elucidating microRNA functions through target prediction. Nucleic Acids Res 37: W273-276.
3. Betel D, Koppal A, Agius P, Sander C, Leslie C (2010) Comprehensive modeling of microRNA targets predicts functional non-conserved and non-canonical sites. Genome Biol 11: R90.
4. Krek A, Grun D, Poy MN, Wolf R, Rosenberg L, et al. (2005) Combinatorial microRNA target predictions. Nat Genet 37: 495-500.
5. Miranda KC, Huynh T, Tay Y, Ang YS, Tam WL, et al. (2006) A pattern-based method for the identification of MicroRNA binding sites and their corresponding heteroduplexes. Cell 126: 1203-1217.
6. Rehmsmeier M, Steffen P, Hochsmann M, Giegerich R (2004) Fast and effective prediction of microRNA/target duplexes. RNA 10: 1507-1517.
7. Lewis BP, Shih IH, Jones-Rhoades MW, Bartel DP, Burge CB (2003) Prediction of mammalian microRNA targets. Cell 115: 787-798.
8. Lewis BP, Burge CB, Bartel DP (2005) Conserved seed pairing, often flanked by adenosines, indicates that thousands of human genes are microRNA targets. Cell 120: 15-20.
9. Kertesz M, Iovino N, Unnerstall U, Gaul U, Segal E (2007) The role of site accessibility in microRNA target recognition. Nat Genet 39: 1278-1284.
10. Wang X, El Naqa IM (2008) Prediction of both conserved and nonconserved microRNA targets in animals. Bioinformatics 24: 325-332.
11. Wang X (2008) miRDB: a microRNA target prediction and functional annotation database with a wiki interface. RNA 14: 1012-1017.
12. Bandyopadhyay S, Mitra R (2009) TargetMiner: microRNA target prediction with systematic identification of tissue-specific negative examples. Bioinformatics 25: 2625-2631.
13. John B, Enright AJ, Aravin A, Tuschl T, Sander C, et al. (2004) Human MicroRNA targets. PLoS Biol 2:

e363.
14. Enright AJ, John B, Gaul U, Tuschl T, Sander C, et al. (2003) MicroRNA targets in Drosophila. Genome Biol 5: R1.
